# Sex-Related Differences in the Associations between Adiponectin and Serum Lipoproteins in Healthy Subjects and Patients with Metabolic Syndrome

**DOI:** 10.3390/biomedicines12091972

**Published:** 2024-09-01

**Authors:** Iva Klobučar, Hansjörg Habisch, Lucija Klobučar, Matias Trbušić, Gudrun Pregartner, Andrea Berghold, Gerhard M. Kostner, Hubert Scharnagl, Tobias Madl, Saša Frank, Vesna Degoricija

**Affiliations:** 1 Department of Cardiology, Sisters of Charity University Hospital Centre, 10000 Zagreb, Croatia; iva.klobucar@gmail.com (I.K.); matias.trbusic@gmail.com (M.T.); 2 Otto Loewi Research Center, Medicinal Chemistry, Medical University of Graz, 8010 Graz, Austria; hansjoerg.habisch@medunigraz.at (H.H.); tobias.madl@medunigraz.at (T.M.); 3 Department of Medicine, University Hospital Centre Osijek, 31000 Osijek, Croatia; klobucar.lucija@gmail.com; 4 School of Medicine, University of Zagreb, 10000 Zagreb, Croatia; vesna.degoricija@mef.hr; 5 Institute for Medical Informatics, Statistics, and Documentation, Medical University of Graz, 8036 Graz, Austria; gudrun.pregartner@medunigraz.at (G.P.); andrea.berghold@medunigraz.at (A.B.); 6 Gottfried Schatz Research Center, Molecular Biology and Biochemistry, Medical University of Graz, 8010 Graz, Austria; gerhard.kostner@medunigraz.at; 7 Clinical Institute of Medical and Chemical Laboratory Diagnostics, Medical University of Graz, 8036 Graz, Austria; hubert.scharnagl@medunigraz.at; 8 BioTechMed-Graz, 8010 Graz, Austria; 9 Department of Medicine, Sisters of Charity University Hospital Centre, 10000 Zagreb, Croatia

**Keywords:** adiponectin, lipoprotein subclasses, metabolic syndrome, sex-related differences, NMR spectroscopy

## Abstract

The strong associations between the serum levels of adiponectin and the lipoprotein subclasses observed in healthy subjects are much weaker in patients with metabolic syndrome (MS). However, the impact of sex on these associations remained unexplored. Therefore, in the present study, we examined associations between adiponectin and the lipoprotein subclasses, analyzed by nuclear magnetic resonance spectroscopy, separately in healthy females and males, as well as in females and males with MS. We observed negative correlations between adiponectin and VLDL, IDL, and small-dense LDL in healthy males, but neither in healthy females nor in females or males with MS. Additionally, adiponectin was positively correlated with some HDL subclasses in healthy males and females with MS, but not in healthy females or males with MS. Adjusting for age and either body mass index, waist circumference, C-reactive protein, or interleukin-6 weakened the associations between adiponectin and VLDL and IDL but not small-dense LDL. The adjustment weakened the associations between adiponectin and HDL in healthy males but not in females with MS. Based on our results, we conclude that sex and the presence of MS are strong determinants of the associations between adiponectin and serum lipoproteins and that the complex regulatory network comprising adiponectin and other molecular players involved in the regulation of lipoprotein metabolism is primarily operative in healthy males and females with MS.

## 1. Introduction

Adiponectin is a secretory protein synthesized in adipocytes that is involved in the regulation of glucose, lipid, and lipoprotein metabolism [1,2]. The insulin-sensitizing, anti-inflammatory, and anti-atherogenic activities of adiponectin, revealed by studies in animal models and humans, highlight an important role of adiponectin in the maintenance of cardiovascular and metabolic homeostasis [3,4,5]. Serum levels of adiponectin are inversely correlated with adipose tissue mass and have been found to be decreased in obesity, as well as in insulin resistance, type 2 diabetes mellitus, and metabolic syndrome (MS) [2,6,7,8].

MS is a pathophysiological constellation characterized by abdominal obesity, insulin resistance, hyperglycemia, and high blood pressure, as well as dyslipidemia with increased serum triglycerides (TGs) and small-dense low-density lipoprotein (LDL) but decreased high-density lipoprotein (HDL) [9,10]. MS dyslipidemia is a consequence of an oversupply of the liver with free fatty acids released from inflamed, insulin-resistant visceral adipose tissue and, in consequence, increased production of TG-enriched VLDL, LDL, and HDL [11,12]. Hepatic lipase (HL)-mediated lipolysis of the TG-enriched HDL and LDL generates rapidly catabolizing small-dense HDL and atherogenic small-dense LDL, the hallmarks of MS dyslipidemia [11,12].

Through upregulation of lipoprotein lipase (LPL) and downregulation of HL, adiponectin promotes a lowering of the serum TG levels and, in turn, impedes the generation of the atherogenic small-dense LDL and rapidly catabolizing small-dense HDL [13,14,15,16,17,18]. The positive association between HDL and adiponectin [19,20,21,22,23,24] and a marked overlap of cardiovascular protective and metabolic activities of HDL and adiponectin suggest their mutual regulation and a possibility that adiponectin mediates some physiological effects of HDL and vice versa [1,13,25,26]. 

We have recently reported that the strong associations we observed between adiponectin and the lipoprotein subclasses in healthy subjects were much weaker in patients with MS [27]. The serum levels of adiponectin and lipoproteins differ between males and females, as well as between healthy subjects and patients with MS, as shown in previous reports [1,27,28,29,30,31]. However, no study examined whether the associations between adiponectin and the lipoprotein subclasses differ between females and males and whether the sex-specific differences are affected by MS. 

Therefore, in the present study, we examined associations between adiponectin and the lipoprotein subclasses separately in healthy females and males, as well as in females and males with MS. 

## 2. Materials and Methods

### 2.1. Study Design, Participants, and Routine Laboratory Procedures

The study was a cross-sectional investigation of demographic, clinical, and laboratory parameters in 65 healthy subjects and 65 individuals with MS. MS was defined according to the joint statement given by multiple international professional societies in 2009 [10]. Inclusion and exclusion criteria, as well as all study procedures, have been described in detail in our previous reports [27,32,33,34]. The study was approved by the local ethics committees of the Sisters of Charity University Hospital Centre, Zagreb, Croatia (EP 13125/17-4); the University of Zagreb, School of Medicine, Croatia; and the Medical University of Graz, Austria (31-532 ex 18/19)**.** All participants signed an informed consent, and the study was performed in accordance with the principles of Good Clinical Practice Guidelines and the Declaration of Helsinki [35].

### 2.2. Adiponectin Measurements

Adiponectin levels were measured in undiluted serum using a latex-enhanced turbidimetric immunoassay (2.3 µL serum + 120 µL reagent 1 + 30 µL reagent 2) (Denka Co., Ltd., Tokyo, Japan) on a Beckman Coulter AU680 analyzer (Beckman Coulter, Krefeld, Germany), as reported previously [27].

### 2.3. Lipoprotein Profiling Using Nuclear Magnetic Resonance (NMR) Spectroscopy

Serum lipoprotein subclasses were measured on a Bruker 600 MHz Avance Neo NMR spectrometer (Bruker, Rheinstetten, Germany) using the Bruker IVDr lipoprotein subclass analysis protocol, as described previously [27,32,33]. 

### 2.4. Statistics

Qualitative patient characteristics were summarized using absolute and relative frequencies. Depending on the data distribution, quantitative variables were summarized using mean and standard deviations (SDs) or medians and interquartile ranges (q1, q3). Differences between the sexes were assessed using Fisher’s exact test, t-test, or Mann–Whitney U test, respectively. Correlation analyses using Spearman’s correlation coefficient were performed separately for healthy females and males, as well as females and males with MS (four groups). Partial correlation analyses were performed to examine the impact of confounders using the following four models: Model 1—age and body mass index (BMI); Model 2—age and waist circumference; Model 3—age and C-reactive protein (CRP); Model 4—age and interleukine-6 (IL-6).

A *p*-value of <0.05 was considered significant for the analyses regarding differences in the demographic and clinical characteristics, standard laboratory data, as well as correlation analyses between adiponectin and clinical and laboratory parameters. However, when assessing differences in the serum levels of the lipoprotein subclasses between the study groups, a Bonferroni correction (0.05/95) was applied to correct for multiple testing, and, thus, a *p*-value < 0.0005 was considered significant. Due to the generally small sample size and slightly different numbers of females and males in the four individual groups (N = 31 for females, N = 34 for males), we considered effect sizes rather than *p*-values to interpret the results of the correlation analyses. We therefore considered Spearman correlation coefficients with |r| ≥ 0.5 as ‘’pronounced’’ associations. R version 4.1.0 was used for these analyses.

## 3. Results

### 3.1. Differences in Demographic and Clinical Characteristics between Females and Males within the Groups

While females and males with MS were similar regarding age, BMI, and waist circumference, healthy males were significantly older and had significantly greater BMI and waist circumference compared to healthy females. There were no sex differences within either group regarding heart rate, systolic, diastolic, and mean arterial blood pressure, as well as the frequency of physical activity per week. The incidence of diabetes mellitus type 2 was significantly higher in females with MS, whereas the incidence of hypertension was similar in both sexes (Table 1).

### 3.2. Sex Differences in Serum Levels of Adiponectin, Routine Laboratory Data, and Lipoprotein Subclasses within Each Group

While serum levels of adiponectin were significantly lower, that of glucose, bilirubin, alanine aminotransferase, and gamma-glutamyl transpeptidase (GGT), as well as urea, urate, creatinine, and potassium, were significantly higher in healthy males compared to healthy females. Compared to females with MS, males with MS had significantly lower serum levels of adiponectin, IL-6, and alkaline phosphatase; however, they had significantly higher serum levels of bilirubin, GGT, creatine kinase, urate, and creatinine (Table 2). Original data related to the results shown in Table 1 and Table 2 are presented in Table S1. 

In the MS group, there were no sex differences in the serum levels of the lipoproteins. In contrast, healthy males showed significantly higher values of the indicators of the serum levels of small-dense LDL (serum levels of total cholesterol (TC), free cholesterol (FC), and phospholipids (PLs) in LDL subclasses 5 and 6, as well as serum levels of apoB in LDL subclass 5) than healthy females. Additionally, healthy males had significantly lower serum levels of TG in LDL subclass 2, as well as in HDL subclasses 1 and 2 (Table S2).

### 3.3. Correlation Analyses between Adiponectin and the Serum Levels of the VLDL Subclasses

**Figure 1:**
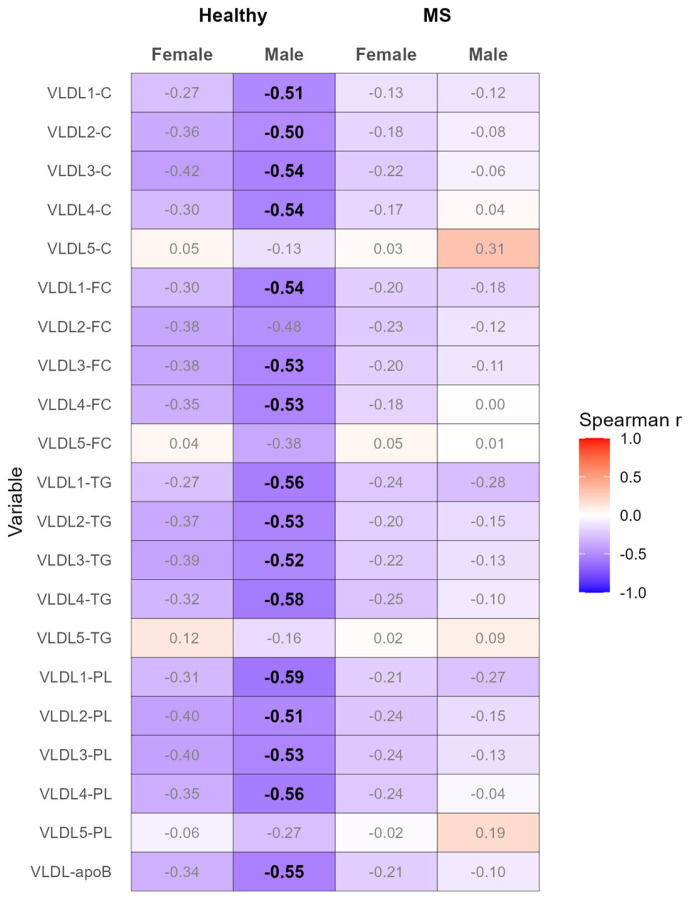
Heatmap of correlation analyses between the serum levels of adiponectin and the VLDL subclasses, performed separately for healthy females and males, as well as females and males with MS. Spearman correlation coefficients with |r| ≥ 0.5 are depicted in bold. ApoB, apolipoprotein B; C, cholesterol; FC, free cholesterol; PL, phospholipid; TG, triglyceride; VLDL, very low-density lipoprotein.

Strikingly, adiponectin was profoundly (negatively) correlated with various indicators of the serum levels of VLDL, namely the serum levels of apolipoprotein B (apoB) in total VLDL, as well as the serum levels of TC, FC, TG, and PL in VLDL subclasses 1–4 (with the exception of serum levels of FC in VLDL subclass 2) in healthy males, but neither in healthy females nor in females or males with MS (Figure 1 and Table S3). Adjusting for age and BMI (Model 1), age and waist circumference (Model 2), age and CRP (Model 3), or age and IL-6 (Model 4) weakened all the pronounced correlations, resulting in coefficients with |r|< 0.5 (Table S4).

### 3.4. Correlation Analyses between Adiponectin and the Serum Levels of IDL 

**Figure 2:**
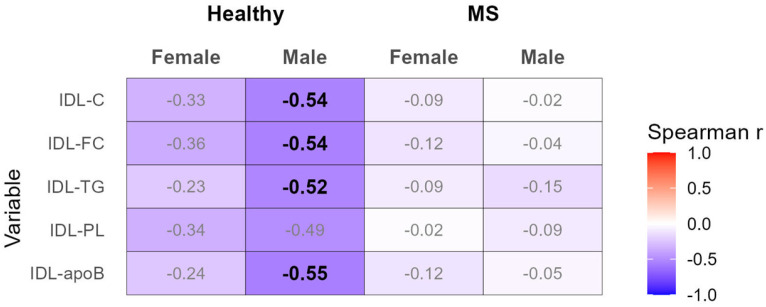
Heatmap of correlation analyses between the serum levels of adiponectin and IDL, performed separately for the healthy females and males, as well as females and males with MS. Spearman correlation coefficients with |r| ≥ 0.5 are depicted in bold. ApoB, apolipoprotein B; C, cholesterol; FC, free cholesterol; IDL, intermediate-density lipoprotein; PL, phospholipid; TG, triglyceride.

Similarly to VLDL, we observed pronounced correlations between adiponectin and the serum levels of TC, FC, TG, and apoB in IDL only in healthy males but not in the other three groups (Figure 2 and Table S3). The pronounced correlations were weakened after adjusting for Models 1–4 (Table S4).

### 3.5. Correlation Analyses between Adiponectin and the Serum Levels of the LDL Subclasses

**Figure 3:**
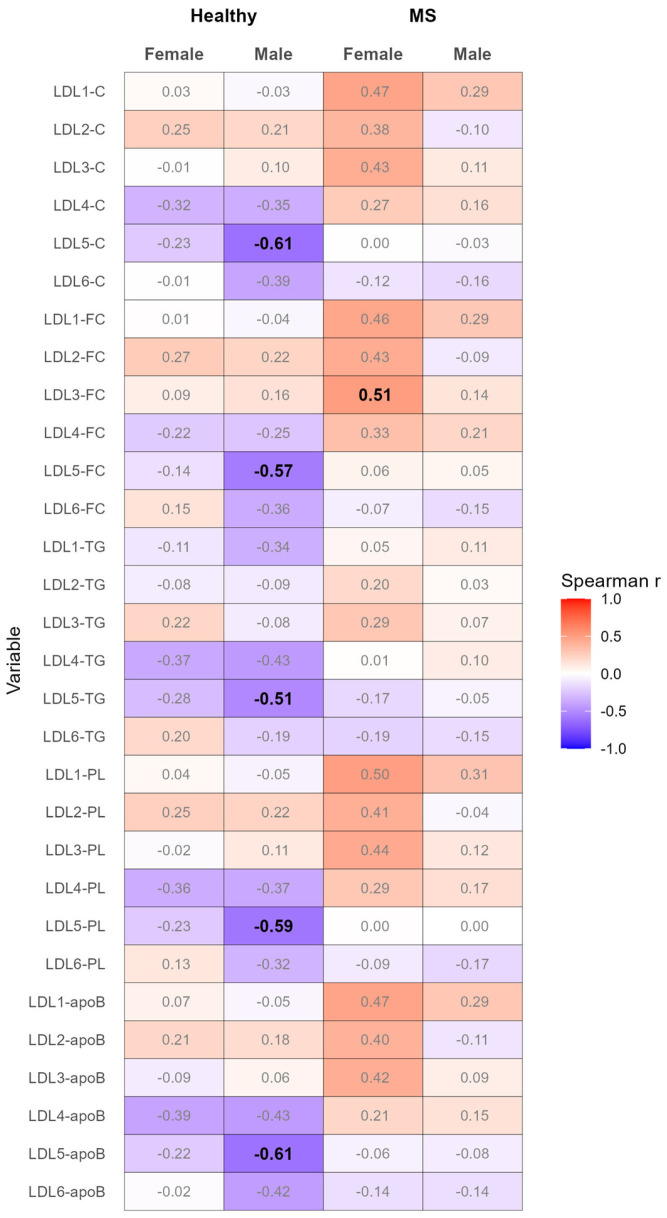
Heatmap of correlation analyses between the serum levels of adiponectin and the LDL subclasses, performed separately for healthy females and males, as well as females and males with MS. Spearman correlation coefficients with |r| ≥ 0.5 are depicted in bold. ApoB, apolipoprotein B; C, cholesterol; FC, free cholesterol; LDL, low-density lipoprotein; PL, phospholipid; TG, triglyceride.

We observed pronounced negative correlations between adiponectin and the serum levels of TC, FC, TG, PL, and apoB in LDL subclass 5 in healthy males but not in the other groups (Figure 3 and Table S3). The strength of these correlations, with the exception of the correlation between adiponectin and the serum levels of TG in LDL subclass 5, was not profoundly affected by adjusting for Models 1–4 (Table S4). Additionally, we found a pronounced positive correlation between adiponectin and the serum levels of FC in LDL subclass 3; however, it was only in females with MS. This association was weakened after adjusting for age and BMI or waist circumference; however, it was slightly strengthened after adjusting for age and CRP (Table S4).

### 3.6. Correlation Analyses between Adiponectin and the Serum Levels of the HDL Subclasses

**Figure 4:**
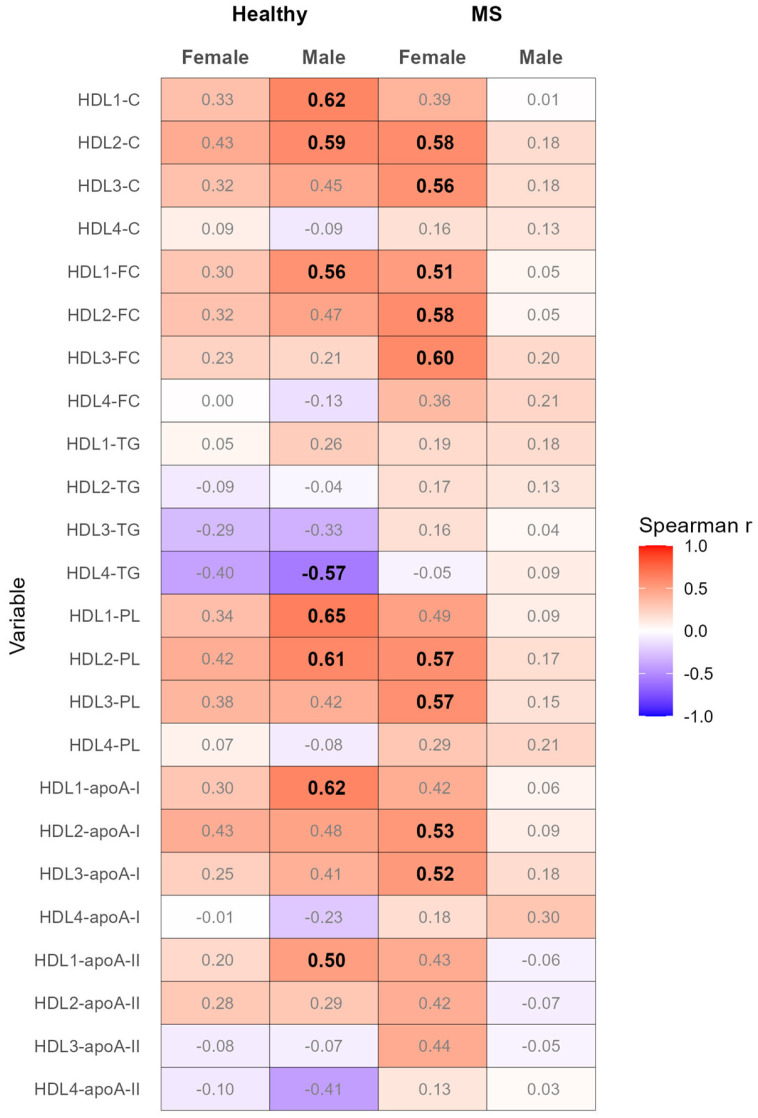
Heatmap of correlation analyses between the serum levels of adiponectin and the HDL subclasses, performed separately for the healthy females and males, as well as females and males with MS. Spearman correlation coefficients with |r| ≥ 0.5 are depicted in bold. ApoB, apolipoprotein B; C, cholesterol; FC, free cholesterol; HDL, high-density lipoprotein; PL, phospholipid; TG, triglyceride.

In healthy males, but not in healthy females, the serum levels of adiponectin were profoundly positively correlated with the serum levels of TC, FC, PL, apoA-I, and apoA-II in HDL subclass 1, as well as with the serum levels of TC and PL in HDL subclass 2 (Figure 4 and Table S3). Additionally, a pronounced negative correlation was observed between adiponectin and the serum levels of TG in HDL subclass 4. However, these correlations (with the exception of adiponectin and HDL1-PL adjusted for Model 1) had a coefficient with |r| < 0.5 after adjusting for Models 1–4 (Table S4). 

In females with MS, but not in males, adiponectin was profoundly positively correlated with the serum levels of TC, FC, PL, and apoA-I in HDL subclasses 2 and 3, respectively, as well as with the serum levels of FC in HDL subclass 1 (Figure 4 and Table S3). Strikingly, the majority of the pronounced correlations (with the exception of the correlations between adiponectin and HDL1-FC and HDL3-apoA-I) were not considerably weakened upon adjusting for Models 1 and 2, and all pronounced correlations were even strengthened after adjusting for Models 3 and 4 (Table S4). 

## 4. Discussion

This article presents, for the first time, that the associations between adiponectin and lipoprotein subclasses markedly differ by sex in healthy subjects, as well as in patients with MS. We also observed sex differences in the associations between adiponectin and several clinical and routine laboratory parameters (Table S5). Our findings are in accordance with established differences between males and females regarding their physiology and metabolic homeostasis [36,37,38,39,40,41,42]. In line with the previously observed attenuation of adiponectin expression by androgens [28], we found lower adiponectin levels in healthy males, as well as males with MS, compared with respective females (Table 2). Age-dependent decline in androgens might be a driver for the positive association between adiponectin and age, which, in the present study, was observed only in healthy males but not in males with MS (Table S5). The differences in the fat tissue distribution (highlighted by predominantly subcutaneous fat in the gluteofemoral region in females and visceral fat in males) and activity (highlighted by the release of free fatty acids, inflammatory cytokines, and adipokines predominantly by visceral adipose tissue) are established causes for the differences in adiponectin levels and energy and lipoprotein metabolism, as well as susceptibility for metabolic disorders between females and males [43,44,45,46,47]. These, together with metabolic perturbations driven by MS pathophysiology, shape the relationships between adiponectin and the serum lipoproteins observed in the present study. 

In the present study, the pronounced negative associations between adiponectin and VLDL subclasses were observed only in healthy males but not in healthy females or females and males with MS. These associations were weakened or abolished after adjustment for age and BMI, waist circumference, IL-6, or CRP, suggesting a role of these confounders in the regulation of adiponectin and VLDL. However, in healthy males, adiponectin and VLDL were profoundly associated only with age but not with BMI, waist circumference, or indicators of inflammation, IL-6 or CRP (Tables S5–S10). The mechanism of adiponectin-mediated induction of VLDL catabolism is a complex interplay of molecular events; it comprises upregulation of LPL and VLDL receptors [23,24,48,49], as well as attenuation of free fatty acid supply to the liver through diminishing free fatty acid release from adipocytes and induction of free fatty acid uptake by skeletal muscle [24,50,51]. Accordingly, it seems that only the physiological environment in healthy males, but not females, supports the regulation of VLDL metabolism by adiponectin. 

In the present study, the pronounced negative associations between adiponectin and the indicators of small-dense LDL observed only in healthy males remained pronounced after adjusting for age and the indicators of adipose tissue mass or inflammation. Remodeling of the large-buoyant LDL to small-dense LDL is determined by the bioavailability of large-buoyant VLDL, as well as the activities of cholesterol ester transfer protein (which, by exchanging triglycerides from VLDL with cholesterol ester of LDL and HDL, catalyzes TG-enrichment of LDL and HDL) and HL (which generates small-dense LDL by cleaving triglyceride-enriched large-buoyant LDL) [11,30,52,53]. Given that HL activity in normolipidemic men is twice the HL activity of women [54,55] and that HL activity is lowered by adiponectin [23,24,48,49], our results suggest that appropriate levels of adiponectin in a physiological environment, as encountered in healthy males, are a prerequisite for efficient regulation of small-dense LDL generation by adiponectin.

It is well documented that estrogen exerts a strong impact on lipid and lipoprotein metabolism [45,56,57,58]. Although the proportion of pre-menopausal females was low in the present study, the lack of association between adiponectin and small-dense LDL in both healthy females as well as females with MS might reflect the interference of estrogen with the action of adiponectin. 

It is well established that adiponectin exerts a positive effect on HDL bioavailability through the promotion of biogenesis and attenuation of the catabolism of HDL [21,22,59]. We observed pronounced positive associations between adiponectin and the indicators of large-buoyant HDL (HDL subclasses 1 and 2) in healthy males but not females, as well as with HDL subclasses 2 and 3 in females but not males, with MS (Table S3, Figure 4). Interestingly, while the pronounced associations between adiponectin and HDL in healthy males were markedly weakened, those in females with MS were not or were even strengthened after adjustments for age and the indicators of adipose tissue mass or inflammation (Table S4). It seems that in healthy males, the increasing age, which is positively associated with both adiponectin (Table S5) and large-buoyant HDL (Table S6), promotes the positive associations between adiponectin and large-buoyant HDL. In females with MS, however, neither adiponectin nor HDL were associated with age or with the indicators of adipose tissue mass or inflammation (Tables S5–S10). Accordingly, it appears that the hormonal and pathophysiological constellation in females with MS facilitates the augmenting effect of adiponectin on HDL bioavailability, exemplified by the observed positive associations between adiponectin and HDL, independently of the tested confounders. This implies that the impact of confounders, which modulate the bioavailability of adiponectin, inflammatory cytokines, and lipoproteins [1,7,29,31,43,60], on the association of adiponectin with HDL is sex-dependent and modulated by the presence of MS. 

Our results clearly show that the impact of adiponectin on serum lipoprotein levels is strongly modulated by sex and that this impact of sex is different in healthy subjects and patients with MS. Consequently, our results suggest that lifestyle and pharmacological intervention for boosting adiponectin levels to improve serum lipoprotein status would be successful primarily in healthy males, but not females, as well as in females, but not males with MS. This knowledge may help design sex-specific therapeutic strategies for treatment of cardio-metabolic diseases caused by low adiponectin and pro-atherogenic lipoprotein profiles. 

Strengths and limitations: The strength of our study is the comprehensive analysis of the serum levels of lipoprotein subclasses in healthy subjects and patients with MS. This enabled comprehensive analyses of the associations between the lipoprotein parameters and adiponectin in the study groups. However, due to the design of the present study, we could not examine causality for the relationship between adiponectin and the lipoprotein subclasses. Accordingly, we could not examine the mechanistic relationship between adiponectin and the lipoprotein subclasses. Additionally, due to the generally small sample size and slightly different numbers of females and males in the groups, we could not consider *p*-values to interpret the results of the correlation analyses between adiponectin and lipoproteins. Even though the majority of pronounced associations exhibited very low *p*-values, only some of them reached statistical significance after a Bonferroni correction for multiple testing. However, since we focused on effect sizes in the correlation analyses rather than mere *p*-values, the sample size issue appeared acceptable. We only considered rather large (or ‘’pronounced’’, |r| ≥ 0.5) associations within the small subgroups relevant enough to mention. Furthermore, due to a low proportion of pre-menopausal females, we could not address the possible impact of estrogen on the associations between adiponectin and lipoproteins. Also, when interpreting the strikingly different values for healthy females and females with MS, we need to keep in mind that twice as many females with MS were post-menopausal as compared to healthy females. It is generally accepted that the term gender refers to the socially constructed norms that impose and determine roles, relationships, and power for all people across their lifetime. In contrast, sex refers to the biological and physical characteristics that define females, males, and those with intersex identities [37,61]. Since the focus of the present study was to analyze differences between biologically defined females and males, and due to the lack of data on the study participants’ genders, in this paper, we analyzed and reported exclusively sex- but not gender-related differences and relationships. Thus, we consider our work an important contribution to sex-related differences in human (patho)physiology that warrants further studies in larger cohorts and different ethnic groups to confirm the concepts of the present study.

## 5. Conclusions

Based on our results, we conclude that sex and the presence of MS are strong determinants of the associations between adiponectin and serum lipoproteins. It appears that the complex regulatory network comprising adiponectin and other molecular players involved in the regulation of lipoprotein metabolism is primarily operative in healthy males and in females with MS. 

Our study provides additional new evidence on the pronounced metabolic differences between females and males under healthy and pathophysiological conditions, thus highlighting the importance of performing pre-clinical and clinical research in both sexes to elucidate disparities in (patho)physiology, disease susceptibility and progression, clinical signs and symptoms, and response to treatment [31,37,46,62].

## Figures and Tables

**Table 1:** Differences in clinical parameters between healthy females and males, as well as females and males with MS.

	Healthy			MS		
Variable	Female (N = 31)	Male (N = 34)	*p*-Value	Female (N = 31)	Male (N = 34)	*p*-Value
Age (years)	52.0 (47.0, 57.0)	57.0 (53.8, 61.0)	**0.002**	56.0 (49.0, 60.0)	57.50 (52.0, 60.0)	0.808
BMI (kg/m^2^)	24.6 (23.7, 26.0)	27.12 (23.5, 29.2)	**0.037**	32.8 (30.1, 39.8)	32.4 (29.5, 34.8)	0.222
Waist circumference (cm)	85.0 (81.5, 87.0)	98.0 (94.0, 104.8)	**<0.001**	110.0 (103.5, 119.0)	114.0 (107.0, 125.0)	0.193
HR (beats/min)	65.0 (58.5, 70.0)	62.50 (58.0, 69.8)	0.693	72.0 (68.5, 76.0)	73.00 (65.3, 76.0)	0.519
SBP (mm Hg)	120.0 (112.5, 125.0)	125.0 (115.0, 130.0)	0.218	140.0 (130.0, 145.0)	140.0 (130.0, 145.0)	0.685
DBP (mm Hg)	70.0 (70.0, 80.0)	80.0 (70.0, 80.0)	0.136	80.0 (80.0, 80.0)	80.0 (80.0, 80.0)	0.411
MAP (mmHg)	86.7 (84.2, 95.0)	93.3 (85.4, 96.7)	0.125	98.33 (96.7, 101.7)	99.2 (96.7, 103.3)	0.947
**Chronic diseases**						
High blood pressure	0 (0.0%)	0 (0.0%)		29 (93.5%)	31 (91.2%)	0.720
Diabetes mellitus type 2	0 (0.0%)	0 (0.0%)		20 (64.5%)	7 (20.6%)	**<0.001**
**Functions and habits**						
Physical activity (≥3 times/week)	27 (87.1%)	31 (91.2%)	0.596	19 (61.3%)	28 (82.4%)	0.058
Menstrual cycle	12/31 (38.7%)			6/31 (19.4%)		

Data are presented as N (%) or median (q1, q3). Differences between groups were tested using Fisher’s exact test or Mann–Whitney U test, respectively. *p*-values <0.05 are considered statistically significant and are depicted in bold. BMI, body mass index; cm, centimeter; DBP, diastolic blood pressure; HR, heart rate; MAP, mean arterial pressure; MS, metabolic syndrome patient; SBP, systolic blood pressure.

**Table 2:** Differences in laboratory parameters between healthy females and males, as well as females and males with MS.

	Healthy			MS		
Variable	Female (N = 31)	Male (N = 34)	*p*-Value	Female (N = 31)	Male (N = 34)	*p*-Value
Adiponectin (µg/mL)	20.20 (14.75, 25.90)	13.10 (10.00, 16.65)	**<0.001**	14.90 (11.85, 18.50)	10.60 (8.80, 13.82)	**0.002**
TG (mmol/L)	0.99 (0.81, 1.25)	1.11 (0.79, 1.39)	0.442	1.52 (1.05, 2.13)	1.71 (1.16, 2.58)	0.325
TC (mmol/L)	5.41 (5.12, 5.95)	5.62 (5.13, 5.97)	0.660	4.84 (4.26, 5.94)	5.25 (4.32, 6.54)	0.386
LDL-C (mmol/L)	3.04 (2.76, 3.71)	3.35 (2.95, 4.01)	0.279	2.83 (2.33, 3.56)	3.12 (2.39, 3.93)	0.379
HDL-C (mmol/L)	1.66 (1.47, 2.01)	1.50 (1.33, 1.81)	0.084	1.22 (1.12, 1.44)	1.14 (0.91, 1.40)	0.070
Glucose (mmol/L)	4.88 (4.69, 5.13)	5.08 (4.84, 5.49)	**0.027**	5.66 (5.30, 6.60)	5.66 (5.36, 6.33)	0.963
Protein (g/L)	71.00 (69.00, 74.00)	73.00 (69.00, 75.00)	0.418	75.00 (72.00, 77.00)	74.00 (71.00, 77.00)	0.589
Albumin (g/L)	47.00 (46.00, 48.00)	47.50 (46.00, 50.00)	0.235	48.00 (45.50, 49.00)	48.00 (45.00, 49.75)	0.995
CRP (µg/mL)	0.90 (0.70, 1.85)	1.45 (0.54, 2.90)	0.176	2.40 (1.75, 6.75)	2.60 (1.12, 4.25)	0.354
IL-6 (pg/mL)	2.10 (1.36, 2.85)	2.50 (1.90, 3.98)	0.129	6.00 (3.50, 8.10)	3.95 (2.50, 5.60)	**0.008**
Bilirubin (µmol/L)	7.87 (5.47, 11.54)	10.43 (7.87, 15.18)	**0.010**	5.99 (4.62, 8.12)	9.66 (7.35, 11.12)	**0.001**
AST (U/L)	21.00 (18.00, 25.00)	23.00 (21.25, 24.75)	0.116	23.00 (19.00, 32.50)	23.00 (20.00, 30.50)	0.703
ALT (U/L)	19.00 (14.00, 23.00)	24.00 (20.00, 36.00)	0.001	28.00 (20.00, 41.00)	32.50 (23.50, 43.75)	0.210
AP (U/L)	60.00 (51.00, 70.50)	59.00 (48.75, 67.75)	0.870	71.00 (62.00, 84.00)	60.00 (51.00, 67.50)	**0.006**
GGT (U/L)	13.00 (11.00, 16.00)	27.00 (16.25, 41.25)	**<0.001**	23.00 (17.00, 29.00)	37.50 (31.00, 55.25)	**<0.001**
CK (U/L)	93.00 (67.00, 136.00)	126.00 (97.00, 163.50)	0.056	119.00 (70.00, 195.50)	169.50 (98.75, 254.00)	0.033
LDH (U/L)	173.00 (157.00, 188.00)	163.50 (145.50, 191.75)	0.325	178.00 (147.50, 194.50)	174.50 (160.50, 191.25)	0.713
Urea (mmol/L)	4.32 (3.90, 5.06)	5.73 (4.57, 6.31)	**<0.001**	5.48 (4.90, 6.97)	5.73 (4.81, 6.31)	0.608
Urate (µmol/L)	232.05 (199.32, 249.90)	318.32 (285.60, 361.46)	**<0.001**	297.50 (249.90, 321.30)	345.10 (315.35, 377.83)	**0.001**
Creatinine (µmol/L)	69.03 (64.16, 73.90)	88.06 (78.99, 93.81)	**<0.001**	66.38 (61.06, 73.01)	84.08 (78.77, 90.27)	**<0.001**
eGFR (mL/min/1.73 m^2^)	88.30 (78.53, 95.28)	85.86 (75.94, 93.33)	0.537	87.97 (77.23, 99.04)	89.01 (82.74, 97.52)	0.941
Sodium (mmol/L)	140.00 (138.00, 141.50)	140.00 (139.00, 141.00)	0.695	139.00 (137.50, 140.00)	140.00 (138.00, 140.75)	0.378
Potassium (mmol/L)	4.10 (3.95, 4.30)	4.40 (4.20, 4.60)	**0.005**	4.20 (4.10, 4.60)	4.20 (4.03, 4.60)	0.833
Chloride (mmol/L)	101.00 (99.00, 103.00)	101.00 (100.00, 103.00)	0.601	100.00 (97.50, 101.50)	100.00 (98.00, 101.00)	0.953

Data are presented as median (q1, q3). Differences between the groups were tested using the Mann–Whitney U test. *p*-values < 0.05 are considered statistically significant and are depicted in bold. ALT, alanine aminotransferase; AP, alkaline phosphatase; AST, aspartate aminotransferase; CK, creatine kinase; CRP, C-reactive protein; eGFR, estimated glomerular filtration rate; GGT, gamma-glutamyl transpeptidase; HDL-C, high-density lipoprotein cholesterol; IL-6, interleukin-6; LDH, lactate dehydrogenase; LDL-C, low-density lipoprotein cholesterol; MS, metabolic syndrome patient; TC, total cholesterol; TG, triglyceride.

## Data Availability

Original data are available upon request.

## Supplementary Materials

The following supporting information can be downloaded at: https://www.mdpi.com/article/10.3390/biomedicines12091972/s1, Table S1: Original data of clinical and laboratory parameters of the study participants; Table S2: Differences in serum levels of VLDL, IDL, LDL, and HDL between healthy females and males, as well as females and males with MS; Table S3: Correlation analyses of serum levels of adiponectin with serum levels of VLDL, IDL, LDL, and HDL, performed separately in healthy females and males, as well as females and males with MS; Table S4: Partial correlation analyses of serum levels of adiponectin with selected lipoprotein parameters in healthy males and females with MS; Table S5: Correlation analyses between serum levels of adiponectin and clinical and laboratory parameters, performed separately in healthy females and males, as well as females and males with MS; Table S6: Correlation analyses of age with serum levels of VLDL, IDL, LDL, and HDL, performed separately in healthy females and males, as well as females and males with MS; Table S7: Correlation analyses of BMI with serum levels of VLDL, IDL, LDL, and HDL, performed separately in healthy females and males, as well as females and males with MS; Table S8: Correlation analyses of waist circumference with serum levels of VLDL, IDL, LDL, and HDL, performed separately in healthy females and males, as well as females and males with MS; Table S9: Correlation analyses of IL-6 with serum levels of VLDL, IDL, LDL, and HDL, performed separately in healthy females and males, as well as females and males with MS; Table S10: Correlation analyses of CRP with serum levels of VLDL, IDL, LDL, and HDL, performed separately in healthy females and males, as well as females and males with MS.

## Abbreviations

ALT	alanine aminotransferase
Apo	apolipoprotein
AST	aspartate aminotransferase
BMI	body mass index
C	cholesterol
CK	creatine kinase
CRP	C-reactive protein
DBP	diastolic blood pressure
eGFR	estimated glomerular filtration rate
FC	free cholesterol
GGT	gamma-glutamyl transpeptidase
HDL	high-density lipoprotein
HL	hepatic lipase
HR	heart rate
IDL	Intermediate-density lipoprotein
IL-6	Interleukin-6
LDH	lactate dehydrogenase
LDL	low-density lipoprotein
LPL	lipoprotein lipase
MAP	mean arterial pressure
MS	metabolic syndrome
NMR	nuclear magnetic resonance
PL	phospholipid
SBP	systolic blood pressure
TC	total cholesterol
TG	triglyceride
VLDL	very low-density lipoprotein

## References

[1] Christou G.A., Kiortsis D.N. (2013). Adiponectin and lipoprotein metabolism. Obes. Rev..

[2] Yanai H., Hirowatari Y., Yoshida H. (2019). Diabetic dyslipidemia: Evaluation and mechanism. Glob. Health Med..

[3] Yamauchi T., Kamon J., Minokoshi Y., Ito Y., Waki H., Uchida S., Yamashita S., Noda M., Kita S., Ueki K. (2002). Adiponectin stimulates glucose utilization and fatty-acid oxidation by activating AMP-activated protein kinase. Nat. Med..

[4] Yamauchi T., Kamon J., Waki H., Terauchi Y., Kubota N., Hara K., Mori Y., Ide T., Murakami K., Tsuboyama-Kasaoka N. (2001). The fat-derived hormone adiponectin reverses insulin resistance associated with both lipoatrophy and obesity. Nat. Med..

[5] Ouchi N., Kihara S., Arita Y., Nishida M., Matsuyama A., Okamoto Y., Ishigami M., Kuriyama H., Kishida K., Nishizawa H. (2001). Adipocyte-derived plasma protein, adiponectin, suppresses lipid accumulation and class A scavenger receptor expression in human monocyte-derived macrophages. Circulation.

[6] Zocchi M., Della Porta M., Lombardoni F., Scrimieri R., Zuccotti G.V., Maier J.A., Cazzola R. (2022). A Potential Interplay between HDLs and Adiponectin in Promoting Endothelial Dysfunction in Obesity. Biomedicines.

[7] Lara-Castro C., Fu Y., Chung B.H., Garvey W.T. (2007). Adiponectin and the metabolic syndrome: Mechanisms mediating risk for metabolic and cardiovascular disease. Curr. Opin. Lipidol..

[8] Combs T.P., Berg A.H., Obici S., Scherer P.E., Rossetti L. (2001). Endogenous glucose production is inhibited by the adipose-derived protein Acrp30. J. Clin. Investig..

[9] Kahn B.B., Flier J.S. (2000). Obesity and insulin resistance. J. Clin. Investig..

[10] Alberti K.G., Eckel R.H., Grundy S.M., Zimmet P.Z., Cleeman J.I., Donato K.A., Fruchart J.C., James W.P., Loria C.M., Smith S.C. (2009). Harmonizing the metabolic syndrome: A joint interim statement of the International Diabetes Federation Task Force on Epidemiology and Prevention; National Heart, Lung, and Blood Institute; American Heart Association; World Heart Federation; International Atherosclerosis Society; and International Association for the Study of Obesity. Circulation.

[11] Adiels M., Olofsson S.O., Taskinen M.R., Boren J. (2008). Overproduction of very low-density lipoproteins is the hallmark of the dyslipidemia in the metabolic syndrome. Arterioscler. Thromb. Vasc. Biol..

[12] Hirano T. (2018). Pathophysiology of Diabetic Dyslipidemia. J. Atheroscler. Thromb..

[13] Christou G.A., Tellis K.C., Elisaf M.C., Tselepis A.D., Kiortsis D.N. (2012). High density lipoprotein is positively correlated with the changes in circulating total adiponectin and high molecular weight adiponectin during dietary and fenofibrate treatment. Hormones.

[14] Riestra P., Garcia-Anguita A., Lasuncion M.A., Cano B., de Oya M., Garces C. (2011). Relationship of adiponectin with metabolic syndrome components in pubertal children. Atherosclerosis.

[15] Shin M.J., Kim O.Y. (2011). Plasma adiponectin is associated with less atherogenic lipoprotein phenotype. Nutr. Metab. Cardiovasc. Dis..

[16] Tsubakio-Yamamoto K., Sugimoto T., Nishida M., Okano R., Monden Y., Kitazume-Taneike R., Yamashita T., Nakaoka H., Kawase R., Yuasa-Kawase M. (2012). Serum adiponectin level is correlated with the size of HDL and LDL particles determined by high performance liquid chromatography. Metab. Clin. Exp..

[17] Qiao L., Zou C., van der Westhuyzen D.R., Shao J. (2008). Adiponectin reduces plasma triglyceride by increasing VLDL triglyceride catabolism. Diabetes.

[18] Weiss R., Otvos J.D., Flyvbjerg A., Miserez A.R., Frystyk J., Sinnreich R., Kark J.D. (2009). Adiponectin and lipoprotein particle size. Diabetes Care.

[19] Oku H., Matsuura F., Koseki M., Sandoval J.C., Yuasa-Kawase M., Tsubakio-Yamamoto K., Masuda D., Maeda N., Ohama T., Ishigami M. (2007). Adiponectin deficiency suppresses ABCA1 expression and ApoA-I synthesis in the liver. FEBS Lett..

[20] Matsuura F., Oku H., Koseki M., Sandoval J.C., Yuasa-Kawase M., Tsubakio-Yamamoto K., Masuda D., Maeda N., Tsujii K., Ishigami M. (2007). Adiponectin accelerates reverse cholesterol transport by increasing high density lipoprotein assembly in the liver. Biochem. Biophys. Res. Commun..

[21] Chan D.C., Barrett P.H., Ooi E.M., Ji J., Chan D.T., Watts G.F. (2009). Very low density lipoprotein metabolism and plasma adiponectin as predictors of high-density lipoprotein apolipoprotein A-I kinetics in obese and nonobese men. J. Clin. Endocrinol. Metab..

[22] Verges B., Petit J.M., Duvillard L., Dautin G., Florentin E., Galland F., Gambert P. (2006). Adiponectin is an important determinant of apoA-I catabolism. Arterioscler. Thromb. Vasc. Biol..

[23] von Eynatten M., Schneider J.G., Humpert P.M., Rudofsky G., Schmidt N., Barosch P., Hamann A., Morcos M., Kreuzer J., Bierhaus A. (2004). Decreased plasma lipoprotein lipase in hypoadiponectinemia: An association independent of systemic inflammation and insulin resistance. Diabetes Care.

[24] Schneider J.G., von Eynatten M., Schiekofer S., Nawroth P.P., Dugi K.A. (2005). Low plasma adiponectin levels are associated with increased hepatic lipase activity in vivo. Diabetes Care.

[25] Drew B.G., Duffy S.J., Formosa M.F., Natoli A.K., Henstridge D.C., Penfold S.A., Thomas W.G., Mukhamedova N., de Courten B., Forbes J.M. (2009). High-density lipoprotein modulates glucose metabolism in patients with type 2 diabetes mellitus. Circulation.

[26] Kriketos A.D., Gan S.K., Poynten A.M., Furler S.M., Chisholm D.J., Campbell L.V. (2004). Exercise increases adiponectin levels and insulin sensitivity in humans. Diabetes Care.

[27] Klobucar I., Habisch H., Klobucar L., Trbusic M., Pregartner G., Berghold A., Kostner G.M., Scharnagl H., Madl T., Frank S. (2024). Serum Levels of Adiponectin Are Strongly Associated with Lipoprotein Subclasses in Healthy Volunteers but Not in Patients with Metabolic Syndrome. Int. J. Mol. Sci..

[28] Nishizawa H., Shimomura I., Kishida K., Maeda N., Kuriyama H., Nagaretani H., Matsuda M., Kondo H., Furuyama N., Kihara S. (2002). Androgens decrease plasma adiponectin, an insulin-sensitizing adipocyte-derived protein. Diabetes.

[29] Tomono Y., Hiraishi C., Yoshida H. (2018). Age and sex differences in serum adiponectin and its association with lipoprotein fractions. Ann. Clin. Biochem..

[30] Ryo M., Nakamura T., Kihara S., Kumada M., Shibazaki S., Takahashi M., Nagai M., Matsuzawa Y., Funahashi T. (2004). Adiponectin as a biomarker of the metabolic syndrome. Circ. J..

[31] Mester P., Rath U., Schmid S., Muller M., Buechler C., Pavel V. (2023). Exploring the Relationship between Plasma Adiponectin, Gender, and Underlying Diseases in Severe Illness. Biomedicines.

[32] Klobucar I., Klobucar L., Lechleitner M., Trbusic M., Pregartner G., Berghold A., Habisch H., Madl T., Frank S., Degoricija V. (2023). Associations between Endothelial Lipase and Apolipoprotein B-Containing Lipoproteins Differ in Healthy Volunteers and Metabolic Syndrome Patients. Int. J. Mol. Sci..

[33] Klobucar I., Stadler J.T., Klobucar L., Lechleitner M., Trbusic M., Pregartner G., Berghold A., Habisch H., Madl T., Marsche G. (2023). Associations between Endothelial Lipase, High-Density Lipoprotein, and Endothelial Function Differ in Healthy Volunteers and Metabolic Syndrome Patients. Int. J. Mol. Sci..

[34] Klobucar I., Hofmann L., Habisch H., Lechleitner M., Klobucar L., Trbusic M., Pregartner G., Berghold A., Madl T., Frank S. (2024). Advanced Oxidation Protein Products Are Strongly Associated with the Serum Levels and Lipid Contents of Lipoprotein Subclasses in Healthy Volunteers and Patients with Metabolic Syndrome. Antioxidants.

[35] World Medical A. (2013). World Medical Association Declaration of Helsinki: Ethical principles for medical research involving human subjects. JAMA.

[36] Carrero J.J., Hecking M., Chesnaye N.C., Jager K.J. (2018). Sex and gender disparities in the epidemiology and outcomes of chronic kidney disease. Nat. Rev. Nephrol..

[37] Mauvais-Jarvis F., Bairey Merz N., Barnes P.J., Brinton R.D., Carrero J.J., DeMeo D.L., De Vries G.J., Epperson C.N., Govindan R., Klein S.L. (2020). Sex and gender: Modifiers of health, disease, and medicine. Lancet.

[38] Ahonen T., Saltevo J., Laakso M., Kautiainen H., Kumpusalo E., Vanhala M. (2009). Gender differences relating to metabolic syndrome and proinflammation in Finnish subjects with elevated blood pressure. Mediat. Inflamm..

[39] Kautzky-Willer A., Harreiter J., Pacini G. (2016). Sex and Gender Differences in Risk, Pathophysiology and Complications of Type 2 Diabetes Mellitus. Endocr. Rev..

[40] Juszczak F., Pierre L., Decarnoncle M., Jadot I., Martin B., Botton O., Caron N., Dehairs J., Swinnen J.V., Decleves A.E. (2023). Sex differences in obesity-induced renal lipid accumulation revealed by lipidomics: A role of adiponectin/AMPK axis. Biol. Sex Differ..

[41] Palmer B.F., Clegg D.J. (2015). The sexual dimorphism of obesity. Mol. Cell Endocrinol..

[42] Saltevo J., Kautiainen H., Vanhala M. (2009). Gender differences in adiponectin and low-grade inflammation among individuals with normal glucose tolerance, prediabetes, and type 2 diabetes. Gend. Med..

[43] Cnop M., Havel P.J., Utzschneider K.M., Carr D.B., Sinha M.K., Boyko E.J., Retzlaff B.M., Knopp R.H., Brunzell J.D., Kahn S.E. (2003). Relationship of adiponectin to body fat distribution, insulin sensitivity and plasma lipoproteins: Evidence for independent roles of age and sex. Diabetologia.

[44] Gavin K.M., Bessesen D.H. (2020). Sex Differences in Adipose Tissue Function. Endocrinol. Metab. Clin. N. Am..

[45] Palmisano B.T., Zhu L., Eckel R.H., Stafford J.M. (2018). Sex differences in lipid and lipoprotein metabolism. Mol. Metab..

[46] Tramunt B., Smati S., Grandgeorge N., Lenfant F., Arnal J.F., Montagner A., Gourdy P. (2020). Sex differences in metabolic regulation and diabetes susceptibility. Diabetologia.

[47] Wang F.H., Meng L.Y., Yu T.Y., Tan Y., Quan H., Hu J.Y., Bai Q.K., Xie J.C., Zhao Y.X. (2022). Associations of Abdominal Visceral Fat Content and Plasma Adiponectin Level with Intracranial Atherosclerotic Stenosis: A Cross-Sectional Study. Front. Neurol..

[48] Loucif Y., Methot J., Tremblay K., Brisson D., Gaudet D. (2011). Contribution of adiponectin to the cardiometabolic risk of postmenopausal women with loss-of-function lipoprotein lipase gene mutations. Menopause.

[49] Strauss J.G., Frank S., Kratky D., Hammerle G., Hrzenjak A., Knipping G., von Eckardstein A., Kostner G.M., Zechner R. (2001). Adenovirus-mediated rescue of lipoprotein lipase-deficient mice. Lipolysis of triglyceride-rich lipoproteins is essential for high density lipoprotein maturation in mice. J. Biol. Chem..

[50] Brudasca I., Cucuianu M. (2007). Pathogenic role of abnormal fatty acids and adipokines in the portal flow. Relevance for metabolic syndrome, hepatic steatosis and steatohepatitis. Rom. J. Intern. Med..

[51] Ng T.W., Watts G.F., Farvid M.S., Chan D.C., Barrett P.H. (2005). Adipocytokines and VLDL metabolism: Independent regulatory effects of adiponectin, insulin resistance, and fat compartments on VLDL apolipoprotein B-100 kinetics?. Diabetes.

[52] Shirakawa T., Nakajima K., Yatsuzuka S., Shimomura Y., Kobayashi J., Machida T., Sumino H., Murakami M. (2015). The role of circulating lipoprotein lipase and adiponectin on the particle size of remnant lipoproteins in patients with diabetes mellitus and metabolic syndrome. Clin. Chim. Acta Int. J. Clin. Chem..

[53] Lucero D., Zago V., Lopez G.H., Cacciagiu L., Lopez G.I., Wikinski R., Nakajima K., Schreier L. (2012). Predominance of large VLDL particles in metabolic syndrome, detected by size exclusion liquid chromatography. Clin. Biochem..

[54] Tan C.E., Foster L., Caslake M.J., Bedford D., Watson T.D., McConnell M., Packard C.J., Shepherd J. (1995). Relations between plasma lipids and postheparin plasma lipases and VLDL and LDL subfraction patterns in normolipemic men and women. Arterioscler. Thromb. Vasc. Biol..

[55] Carr M.C., Hokanson J.E., Zambon A., Deeb S.S., Barrett P.H., Purnell J.Q., Brunzell J.D. (2001). The contribution of intraabdominal fat to gender differences in hepatic lipase activity and low/high density lipoprotein heterogeneity. J. Clin. Endocrinol. Metab..

[56] Conlon D.M., Welty F.K., Reyes-Soffer G., Amengual J. (2023). Sex-Specific Differences in Lipoprotein Production and Clearance. Arterioscler. Thromb. Vasc. Biol..

[57] Palmisano B.T., Zhu L., Stafford J.M. (2017). Role of Estrogens in the Regulation of Liver Lipid Metabolism. Adv. Exp. Med. Biol..

[58] Holven K.B., Roeters van Lennep J. (2023). Sex differences in lipids: A life course approach. Atherosclerosis.

[59] Rashid S., Watanabe T., Sakaue T., Lewis G.F. (2003). Mechanisms of HDL lowering in insulin resistant, hypertriglyceridemic states: The combined effect of HDL triglyceride enrichment and elevated hepatic lipase activity. Clin. Biochem..

[60] Stadler J.T., Lackner S., Morkl S., Trakaki A., Scharnagl H., Borenich A., Wonisch W., Mangge H., Zelzer S., Meier-Allard N. (2021). Obesity Affects HDL Metabolism, Composition and Subclass Distribution. Biomedicines.

[61] Shannon G., Jansen M., Williams K., Caceres C., Motta A., Odhiambo A., Eleveld A., Mannell J. (2019). Gender equality in science, medicine, and global health: Where are we at and why does it matter?. Lancet.

[62] Roeters van Lennep J.E., Tokgozoglu L.S., Badimon L., Dumanski S.M., Gulati M., Hess C.N., Holven K.B., Kavousi M., Kayikcioglu M., Lutgens E. (2023). Women, lipids, and atherosclerotic cardiovascular disease: A call to action from the European Atherosclerosis Society. Eur. Heart J..

