# Supplementary material for: Sex-Related Differences in the Associations between Adiponectin and Serum Lipoproteins in Healthy Subjects and Patients with Metabolic Syndrome
Source: Biomedicines. 2024 Sep 1;12(9):1972. doi: 10.3390/biomedicines12091972 (PMC11429094; doi:10.3390/biomedicines12091972)
Supplement: Supplementary file 1 [file biomedicines-12-01972-s001.zip › Table S1.pdf]

N	Group	Sex	Age	Heart rate	Systolic	Diastolic	Mean art	Arterial h	Diabetes	Physical acti	Menstr	Adiponectin	HDL (	Glucose	Protein	Albumin	CRP (	IL-6 (p	Bilirubin	AST (	ALT (	AP (U	GGT (	CK (U	LDH	Urea	Urate (	Creatinine	eGFR (r	Na (r	K (mr	Cl (mmol/L)
1	Healthy	female	59	72	110	70	83,3	no	no	yes	no	35,1	2,16	5,22	74	48	2,3	2,9	7,2	21	29	92	74	45	155	4,3	280	66	87,5	143	4,6	102
2	MS	male	60	78	140	80	100,0	yes	yes	yes		10,3	0,94	6,49	78	48	3,0	4,1	8,0	29	33	60	44	447	184	7,5	381	100	70,3	143	4,7	103
3	Healthy	female	59	65	120	70	86,7	no	no	yes	no	33,2	1,66	5,44	72	46	0,8	2,7	13,5	24	21	61	12	65	228	4,0	232	64	92,0	140	4,3	101
4	MS	male	56	66	140	80	100,0	yes	no	yes		10,1	1,43	5,11	70	50	0,1	2,0	10,1	29	36	52	56	408	188	6,0	274	81	93,9	139	4,5	101
5	Healthy	male	65	76	120	80	93,3	no	no	yes		31,7	2,11	4,72	65	44	0,9	0,5	11,6	22	24	59	16	63	154	6,6	387	80	89,3	141	4,5	103
6	MS	male	62	63	115	70	85,0	no	no	yes		10,9	0,88	5,66	65	44	1,2	3,9	11,1	18	23	65	34	169	171	6,3	399	112	60,2	140	5,2	104
7	Healthy	female	57	67	110	70	83,3	no	no	yes	no	11	1,59	5,44	71	48	5,1	4,4	11,6	23	23	60	21	121	173	3,8	381	73	78,5	140	4,5	100
8	MS	female	58	71	140	80	100,0	yes	no	no	no	25,3	2,31	6,22	76	51	1,4	4,5	7,7	24	24	86	23	98	184	6,5	357	73	79,1	155	4,2	109
9	Healthy	male	59	70	130	80	96,7	no	no	no		19,3	1,51	5,11	68	46	1,7	2,2	18,1	22	22	41	17	36	141	5,0	274	79	93,6	141	4	103
10	Healthy	male	62	61	100	60	73,3	no	no	yes		14	1,40	4,83	74	47	3,0	2,8	9,2	54	41	59	36	738	254	5,6	399	75	93,4	140	4	104
11	Healthy	female	57	75	125	70	88,3	no	no	yes	no	22,6	1,38	5,05	69	47	1,3	3,5	2,7	21	20	71	16	102	173	4,3	232	67	87,4	141	4,4	105
12	Healthy	female	45	66	120	70	86,7	no	no	yes	yes	14,4	1,35	4,33	71	46	1,0	0,6	7,4	18	16	48	5	77	147	3,8	250	71	89,3	138	4	100
13	Healthy	male	53	54	100	60	73,3	no	no	yes		11,3	1,04	4,88	73	48	0,1	0,7	6,3	29	36	63	16	100	83	6,3	262	81	95,9	139	4,3	100
14	Healthy	female	56	49	120	70	86,7	no	no	yes	no	18,3	1,95	4,61	69	46	0,8	0,8	11,6	21	22	41	9	257	174	3,8	226	56	100,6	138	3,9	103
15	MS	male	52	75	160	90	113,3	yes	no	yes		7,4	1,30	5,66	80	45	3,8	7,8	6,0	50	72	78	236	125	193	3,7	309	66	105,5	140	4	96
16	Healthy	female	53	62	120	70	86,7	no	no	no	no	22,7	2,03	4,72	67	45	2,9	1,7	7,7	16	12	72	11	44	186	3,7	220	60	100,2	142	3,8	103
17	Healthy	female	48	84	135	80	98,3	no	no	yes	yes	17,5	1,87	5,16	74	48	1,0	4,2	11,5	18	13	43	13	171	245	5,0	196	68	91,6	138	4,1	103
18	MS	male	60	51	120	70	86,7	no	no	yes		19,7	1,14	5,66	74	48	1,0	2,5	9,6	20	22	80	36	89	159	4,5	298	82	88,9	141	5,3	98
19	Healthy	female	52	57	125	80	95,0	no	no	yes	no	22,8	2,31	4,61	69	47	0,7	2,9	10,6	30	25	53	15	140	214	4,6	244	67	90,5	141	3,8	103
20	Healthy	male	58	66	130	80	96,7	no	no	yes		16,8	1,85	5,00	73	47	3,7	5,2	8,4	20	14	47	10	127	145	5,3	363	90	80,6	141	4,4	104
21	MS	female	65	78	140	80	100,0	yes	yes	yes	no	7,2	1,12	8,71	72	49	2,4	2,6	6,3	53	51	154	137	48	127	4,0	315	50	98,2	138	4,7	101
22	MS	male	59	68	125	70	88,3	yes	no	yes		8,6	0,91	5,61	75	49	2,3	2,6	7,4	22	39	39	34	37	109	6,3	315	79	93,6	141	5,3	100
23	MS	male	63	63	140	80	100,0	yes	no	yes		10	1,61	5,77	69	47	3,3	3,5	8,4	29	34	49	61	257	186	6,1	315	73	93,6	137	4,1	100
24	Healthy	female	61	69	130	80	96,7	no	no	yes	no	21,5	1,85	4,61	69	45	0,7	2,1	5,8	15	17	66	12	75	149	4,3	232	65	89,2	142	3,9	105
25	Healthy	female	47	67	130	80	96,7	no	no	yes	yes	36,7	1,85	4,88	68	46	0,8	0,8	7,4	23	19	60	10	50	188	6,6	232	109	52,4	144	4,1	107
26	MS	female	45	81	115	70	85,0	yes	no	yes	yes	17,4	1,17	4,66	71	45	4,4	5,3	2,6	17	20	66	14	51	147	5,5	202	42	118,8	139	3,5	101
27	MS	female	45	72	140	80	100,0	yes	no	yes	yes	24,2	1,27	5,11	75	48	13,1	8,2	6,2	20	29	70	25	77	158	3,5	250	52	111,0	137	3,8	96
28	Healthy	male	52	55	130	85	100,0	no	no	yes		11,6	1,35	4,44	68	47	1,2	2,8	13,5	24	32	67	14	294	192	5,5	298	89	85,1	139	4,2	100
29	MS	male	57	66	125	80	95,0	no	no	no		13,3	0,65	4,83	67	43	1,2	1,8	7,4	17	6	57	19	82	181	5,8	363	97	74,1	140	4,1	105
30	MS	male	45	75	140	90	106,7	yes	yes	yes		5,7	1,40	6,60	76	52	5,5	6,1	15,6	25	56	62	53	79	131	6,0	315	83	97,5	139	3,8	100
31	Healthy	male	61	54	105	70	81,7	no	no	yes		13,7	1,56	4,94	66	44	0,8	2,4	10,1	23	19	41	17	214	197	6,0	381	94	75,4	142	4,4	101
32	MS	female	60	69	140	80	100,0	yes	no	yes	no	16,7	0,91	5,94	69	43	1,9	5,3	1,2	19	17	63	9	133	195	11,0	274	85	64,5	139	4,1	103
33	Healthy	female	62	78	100	70	80,0	no	no	yes	no	14,9	1,25	4,94	67	46	0,2	6,1	5,1	18	13	61	12	76	146	3,2	208	84	64,4	142	4,2	104
34	MS	female	46	75	130	80	96,7	yes	yes	yes	no	16,3	1,25	5,55	80	48	7,0	6,8	3,8	38	34	73	34	248	233	7,0	309	88	68,6	139	4	95
35	MS	female	50	66	125	70	88,3	no	yes	yes	yes	17,1	1,12	5,27	77	49	1,9	2,5	6,5	15	17	44	13	49	121	4,8	274	65	96,3	136	4,3	96
36	Healthy	female	58	51	125	80	95,0	no	no	yes	no	33,9	1,56	4,72	67	40	0,8	0,9	7,4	17	12	64	10	40	122	5,3	190	70	82,8	140	4	102
37	Healthy	female	59	54	130	80	96,7	no	no	yes	no	38	2,16	4,77	74	48	0,2	1,9	8,0	23	23	49	15	124	205	6,8	238	78	72,1	142	4,8	99
38	Healthy	female	57	60	125	70	88,3	no	no	yes	no	14,6	2,00	4,66	77	50	3,4	2,6	10,3	24	18	87	16	149	184	5,6	327	77	74,2	139	3,8	96
39	Healthy	male	57	65	120	70	86,7	no	no	yes		18,5	1,35	5,49	70	46	1,4	2,6	9,9	23	23	51	33	108	165	4,5	274	73	98,2	140	4,5	102
40	MS	male	61	66	130	80	96,7	yes	no	yes		9,8	0,57	8,33	79	48	2,8	0,9	13,5	19	6	43	28	112	248	6,1	369			141	4,6	100
41	MS	male	60	73	155	100	118,3	yes	no	yes		14,6	1,14	6,16	77	48	1,4	3,4	10,4	19	23	33	25	98	171	5,6	351	85	85,6	140	4,7	98
42	MS	female	59	66	130	80	96,7	yes	yes	yes	no	11,7	1,09	6,11	78	47	15,8	9,5	4,4	19	20	83	27	83	178	8,0	452	100	53,3	139	5,2	99
43	MS	male	55	66	130	80	96,7	yes	no	yes		12	1,64	5,61	77	52	2,4	2,7	11,1	31	54	39	31	348	176	6,3	315	102	71,2	138	4,2	99
44	Healthy	female	56	57	130	80	96,7	no	no	yes	no	26,2	2,57	5,55	68	47	0,2	1,0	7,9	25	29	66	10	187	169	5,0	184	63	95,5	138	3,9	97
45	MS	male	52	90	150	100	116,7	yes	no	yes		13	1,38	5,66	77	48	4,8	4,2	7,4	24	30	51	36	214	216	6,0	315	89	85,1	140	4,6	97
46	Healthy	female	48	69	115	70	85,0	no	no	yes	yes	10,7	2,16	4,88	70	46	0,3	1,0	11,1	26	15	43	6	69	232	3,5	184	58	104,8	139	4,3	99
47	MS	male	64	78	145	80	101,7	yes	no	yes		9,9	1,22	5,66	76	49	4,4	4,9	5,1	22	32	68	33	167	173	7,1	351	90	77,3	143	3,8	98
48	MS	male	46	73	120	70	86,7	yes	no	yes		18,1	0,96	4,77	78	52	0,8	1,6	10,4	34	46	97	31	292	200	4,8	357	112	68,0	140	4,1	98
49	Healthy	male	57	72	115	65	81,7	no	no	yes		10,9	1,22	5,49	78	45	3,1	7,2	7,5	25	37	71	30	129	137	6,5	327	84	88,5	138	4,2	100
50	Healthy	male	51	69	130	80	96,7	no	no	yes		10,3	1,35	4,66	72	50	3,4	3,0	5,3	23	21	51	23	141	162	8,3	321	77	99,9	139	4,1	98
51	Healthy	female	46	63	115	70	85,0	no	no	yes	yes	19,9	2,21	4,77	72	50	0,8	1,7	9,6	23	16	54	10	111	185	4,2	155	69	91,5	138	4,3	99

52	Healthy	male	53	61	130	80	96,7	no	no	yes		9,1	1,46	5,49	72	50	5,0	2,3	9,1	23	28	85	39	125	169	6,1	381	88	86,6	141	4,5	101
53	Healthy	female	51	55	115	70	85,0	no	no	yes	no	25,6	2,91	5,16	76	47	1,8	2,8	14,5	29	27	54	15	199	196	4,3	184	69	88,3	139	4,6	99
54	Healthy	female	54	65	120	80	93,3	no	no	no	no	20,2	1,46	4,66	68	47	2,7	4,7	5,1	19	9	87	13	102	207	2,5	196	50	106,0	140	4,3	98
55	MS	male	58	68	130	80	96,7	yes	no	no		8,5	1,59	5,72	76	53	0,7	2,5	20,9	35	52	49	84	78	159	5,3	387	91	79,7	140	4,2	100
56	MS	male	53	63	125	70	88,3	yes	no	yes		4,7	0,86	5,99	78	51	2,3	1,7	13,3	23	28	45	43	161	125	5,1	518	98	75,4	141	4,2	99
57	MS	female	49	74	145	80	101,7	yes	no	yes	no	10,5	1,07	5,55	73	45	7,3	10,1	3,2	26	28	67	39	183	182	4,0	321	61	102,5	138	4,2	99
58	MS	male	45	69	150	80	103,3	yes	no	yes		6,7	0,88	4,66	75	51	3,1	2,2	7,4	128	326	61	93	357	277	6,3	411	89	90,5	139	4	98
59	MS	male	61	54	145	70	95,0	yes	no	no		12,2	1,12	5,05	74	49	0,9	5,7	10,6	19	22	66	31	98	145	4,5	339	68	97,9	140	4,3	97
60	MS	male	45	79	140	80	100,0	yes	no	yes		9,1	0,86	6,99	81	47	3,8	3,4	2,6	20	38	51	49	170	192	6,5	470	83	97,5	139	4	98
61	MS	female	65	70	150	80	103,3	yes	no	yes	no	32,3	2,26	5,88	76	49	1,8	3,5	10,6	35	57	81	31	69	187	7,3	208	66	83,9	143	5	100
62	MS	male	54	60	135	80	98,3	yes	no	yes		14,1	1,17	5,44	73	49	3,0	4,6	15,0	33	43	45	45	214	186	5,6	345	74	99,3	142	4,5	102
63	Healthy	male	45	81	130	80	96,7	no	no	yes		14,3	1,30	5,11	79	51	8,6	6,1	7,5	23	40	106	42	131	142	5,5	339	60	115,3	141	4,1	100
64	Healthy	female	47	71	120	80	93,3	no	no	yes	no	19,7	1,17	4,83	75	49	0,3	1,1	20,3	20	20	72	15	88	170	2,8	184	74	83,0	138	4,2	97
65	Healthy	female	45	72	120	70	86,7	no	no	yes	yes	22,2	1,77	4,88	71	46	1,7	1,7	12,7	14	8	39	11	38	132	4,5	202	67	95,0	139	4,3	99
66	MS	male	58	74	150	80	103,3	yes	yes	yes		20,3	1,85	13,04	71	46	2,8	3,2	20,7	60	88	66	324	259	265	4,6	434	83	89,0	135	4,1	91
67	Healthy	female	51	55	125	80	95,0	no	no	yes	yes	30,8	1,14	5,11	75	44	6,1	2,0	6,0	28	23	138	16	86	174	4,5	244	82	71,4	142	4,9	102
68	MS	male	52	74	140	80	100,0	yes	no	yes		11,9	1,20	5,66	76	49	1,6	4,1	10,4	22	44	82	45	236	172	7,3	298	88	87,2	138	4,6	99
69	MS	male	47	76	155	90	111,7	yes	no	yes		17,8	1,69	5,66	74	52	1,1	4,0	3,2	22	28	60	20	219	189	5,1	280	68	108,1	140	4	100
70	Healthy	male	59	58	125	80	95,0	no	no	yes		9,9	1,69	4,88	70	49	2,4	2,2	9,6	26	24	28	46	173	162	4,5	363	80	93,2	139	4,4	97
71	Healthy	female	56	60	120	70	86,7	no	no	yes	no	14,1	1,48	4,88	80	52	1,9	4,1	9,1	37	26	80	25	560	168	5,3	280	70	83,9	140	4,3	101
72	Healthy	female	45	66	125	70	88,3	no	no	yes	yes	22,7	1,64	5,38	70	46	0,7	2,3	4,3	25	13	60	12	216	162	5,6	214	64	101,5	135	4	97
73	Healthy	male	46	58	125	70	88,3	no	no	no		6,2	1,12	5,55	69	45	1,7	2,3	12,1	24	36	59	74	132	139	4,3	286	93	84,7	136	4,3	98
74	Healthy	female	45	54	105	60	75,0	no	no	no	yes	15,1	1,61	5,05	77	49	1,2	1,1	13,3	19	12	40	13	90	147	5,6	268	86	70,8	136	5,1	100
75	MS	male	58	79	130	90	103,3	yes	no	yes		8,7	0,91	5,66	71	45	9,0	8,8	6,5	17	17	125	38	86	166	4,3	256	81	91,4	138	4,1	103
76	Healthy	female	60	69	105	60	75,0	no	no	no	no	12,8	1,59	5,00	70	47	1,8	2,5	4,4	33	38	55	28	131	188	4,2	268	73	78,0	142	4	104
77	Healthy	male	59	60	130	80	96,7	no	no	yes		20	1,77	4,22	73	48	1,1	2,8	27,5	21	18	79	13	129	198	4,2	375	91	79,1	140	4,7	101
78	Healthy	male	50	61	130	80	96,7	no	no	yes		12,5	1,17	4,83	69	46	0,3	1,2	24,1	23	24	61	14	96	167	4,2	292	76	101,1	140	4,1	104
79	Healthy	male	46	72	125	80	95,0	no	no	yes		9,4	1,40	5,05	68	47	0,3	1,2	10,9	26	43	58	45	190	166	4,8	274	79	102,5	140	4,2	103
80	Healthy	female	45	81	90	60	70,0	no	no	yes	yes	32,4	1,69	4,38	73	49	0,3	1,6	3,9	27	34	54	24	43	160	4,6	250	65	98,2	136	3,8	98
81	MS	female	49	73	130	80	96,7	no	yes	no	no	14,9	1,04	4,88	71	46	2,2	3,3	3,9	24	35	50	16	68	138	5,3	220	62	102,1	136	4,1	99
82	MS	female	65	90	130	80	96,7	yes	yes	yes	no	13,2	1,22	6,77	75	49	0,7	6,3	4,8	21	23	82	21	112	88	7,5	363	73	75,3	141	5,5	103
83	MS	female	65	68	145	80	101,7	yes	yes	yes	no	18,6	1,01	4,94	75	48	4,8	6,9	5,3	19	13	53	24	71	164	7,0	369	84	63,1	135	4,2	97
84	MS	female	59	72	160	80	106,7	yes	yes	no	no	27,6	1,53	7,44	69	44	9,1	8,9	4,4	19	20	78	10	218	249	9,1	250	63	93,5	142	4,1	107
85	MS	male	60	65	140	90	106,7	yes	yes	yes		25,1	1,66	7,66	74	44	0,7	5,7	18,0	39	23	86	154	127	178	5,0	333	71	97,1	134	4,2	97
86	MS	male	63	75	120	80	93,3	yes	no	no		13,1	1,09	5,61	61	40	8,6	7,2	5,5	24	25	60	38	182	172	6,5	280	64	99,3	137	3,7	103
87	MS	male	48	56	130	80	96,7	yes	no	yes		14	0,88	5,22	71	48	0,9	2,1	9,7	15	17	57	24	79	136	4,6	345	80	100,6	137	3,7	100
88	MS	male	49	80	130	80	96,7	yes	yes	yes		6,2	1,01	6,38	77	55	1,1	2,3	7,7	47	56	69	28	323	195	3,7	345	84	93,6	141	4,6	101
89	Healthy	female	45	61	100	60	73,3	no	no	yes	yes	9,6	1,07	4,83	71	50	1,9	2,5	2,7	20	22	70	20	132	160	5,1	232	79	78,5	139	3,8	102
90	MS	female	62	68	140	80	100,0	yes	no	yes	no	10,6	1,72	5,66	74	49	1,9	8,0	5,0	36	56	71	17	267	207	5,5	244	64	90,0	139	4,1	102
91	MS	female	60	81	130	80	96,7	yes	yes	no	no	10,3	1,35	7,60	72	50	0,7	3,6	4,1	15	17	46	26	64	148	7,5	190	83	66,1	141	4,6	102
92	MS	male	61	84	150	70	96,7	yes	yes	no		9,8	1,48	4,66	70	44	32,8	74,0	9,2	23	40	51	22	101	165	9,0	369	87	82,9	138	4,3	105
93	MS	female	64	62	130	80	96,7	yes	yes	yes	no	14,3	1,14	6,99	75	50	0,7	2,7	8,6	23	42	78	43	118	200	5,5	292	65	85,9	139	4,3	101
94	Healthy	male	57	72	125	80	95,0	no	no	yes		13,8	1,33	5,55	76	53	0,4	8,4	10,8	27	39	63	43	167	191	6,3	345	116	60,0	140	5,1	100
95	MS	female	48	77	140	70	93,3	yes	yes	no	yes	12,1	0,96	12,93	77	45	22,2	7,4	4,8	10	11	86	21	37	138	5,5	375	70	88,8	135	4,2	97
96	MS	female	54	87	135	80	98,3	yes	yes	no	no	9,9	0,78	6,38	77	48	27,0	8,9	12,0	41	55	106	122	134	190	5,5	375	54	103,1	136	3,9	95
97	MS	male	55	74	130	80	96,7	yes	no	yes		11,2	1,20	4,38	71	47	1,4	4,0	5,8	23	30	52	117	291	158	4,0	333	89	84,4	136	4	101
98	MS	female	51	78	170	100	123,3	yes	no	yes	no	21,6	1,35	5,55	79	51	3,7	3,4	12,7	32	47	98	88	175	194	4,8	309	66	92,6	139	4,4	101
99	MS	female	55	66	150	80	103,3	yes	yes	no	no	19,4	1,51	5,49	74	45	13,9	11,5	5,5	25	29	88	14	290	242	4,6	303	61	98,3	138	4,2	101
100	MS	male	64	76	130	80	96,7	yes	no	yes		10,2	1,17	5,33	70	45	7,6	6,3	11,3	23	33	57	21	245	169	8,5	405	85	83,2	137	4,6	99
101	Healthy	female	49	63	95	60	71,7	no	no	yes	yes	10,7	1,17	4,33	74	45	0,9	2,4	14,4	17	19	53	39	54	140	4,2	250	56	105,7	138	4,1	102
102	Healthy	female	50	71	130	80	96,7	no	no	yes	no	16,9	1,53	5,16	73	47	0,4	1,9	3,2	16	17	46	14	93	159	5,0	244	71	86,2	140	4,1	103
103	MS	male	60	54	145	80	101,7	yes	yes	yes		17,8	1,12	9,05	71	45	4,7	5,3	9,9	19	29	62	37	129	155	5,5	268	56	107,1	134	4,2	98

104	MS	male	51	98	210	100	136,7	yes	no	no		8	0,73	7,44	71	41	8,9	6,4	6,2	29	30	95	77	184	218	4,8	607	92	82,7	141	4,2	104
105	Healthy	male	64	64	115	70	85,0	no	no	yes		6,8	1,82	4,83	73	51	0,4	2,6	9,4	26	22	44	24	245	223	9,6	292	92	75,5	142	4,8	103
106	MS	female	64	65	130	80	96,7	yes	yes	yes	no	30,2	2,24	5,27	70	50	0,4	127,0	6,7	37	79	50	21	135	196	5,8	298	65	85,9	138	3,2	95
107	Healthy	male	51	84	130	80	96,7	no	no	yes		9,9	1,33	4,83	75	50	2,0	2,6	17,1	24	37	65	28	115	109	3,7	286	81	97,2	141	4,5	104
108	MS	female	47	69	140	80	100,0	yes	no	yes	no	18,4	1,69	4,94	73	46	6,5	6,0	5,8	29	38	68	38	284	238	5,1	286	53	108,9	138	4,2	98
109	Healthy	male	57	61	130	80	96,7	no	no	yes		11,6	1,95	5,55	74	48	0,6	1,8	18,1	32	29	73	28	186	240	6,0	309	89	83,2	138	4,4	98
110	Healthy	male	56	54	110	80	90,0	no	no	yes		8,4	1,33	5,27	70	49	0,4	1,9	15,6	21	27	68	49	112	150	5,3	315	83	90,3	137	4,2	101
111	Healthy	male	61	62	100	60	73,3	no	no	yes		11,6	1,25	4,94	77	46	14,1	1,7	7,4	20	20	59	26	153	195	8,6	298	94	75,4	141	4,6	103
112	Healthy	male	60	57	125	70	88,3	no	no	yes		16,2	1,72	5,55	75	42	1,4	4,4	7,4	20	16	48	22	91	147	5,8	327	96	74,2	132	3,8	98
113	Healthy	male	56	54	120	70	86,7	no	no	yes		7,4	1,51	5,16	79	50	1,3	1,9	13,0	20	21	55	20	100	179	6,1	250	95	77,2	137	4,4	100
114	MS	female	47	74	140	70	93,3	yes	yes	no	yes	8,9	1,27	4,11	70	47	1,8	3,5	9,7	18	23	53	17	122	136	4,0	292	50	111,4	137	4,6	100
115	Healthy	male	62	62	115	70	85,0	no	no	yes		24,2	2,81	5,22	69	47	0,5	1,2	32,3	19	18	45	12	115	212	4,0	202	77	92,5	138	4,6	100
116	Healthy	male	62	63	120	80	93,3	no	no	yes		14,9	1,85	5,49	77	51	3,6	2,3	5,6	24	39	86	83	81	128	4,2	357	104	66,4	141	4,6	100
117	MS	female	50	69	135	80	98,3	yes	no	yes	no	13,2	1,22	5,61	70	49	3,9	4,1	10,1	44	84	61	26	96	183	5,0	315	70	87,6	140	4,3	101
118	Healthy	male	64	67	130	70	90,0	no	no	yes		15,7	1,72	5,55	73	46	2,2	3,9	7,7	24	29	78	25	88	160	6,1	232	81	87,6	142	4,6	102
119	MS	female	45	78	150	80	103,3	yes	no	no	yes	17,2	2,08	5,66	74	45	5,8	2,2	4,8	23	19	58	17	208	179	5,1	250	73	85,4	137	4	100
120	Healthy	male	62	58	110	60	76,7	no	no	yes		17	1,95	5,00	67	43	1,7	1,9	6,2	23	18	37	10	153	172	6,0	399	90	78,4	142	4,7	105
121	MS	female	56	75	145	80	101,7	yes	yes	no	no	12	1,17	6,60	77	50	2,0	3,9	6,5	33	40	67	27	119	175	4,5	226	67	88,0	142	4,7	102
122	MS	female	55	72	150	90	110,0	yes	yes	yes	no	14	1,12	6,60	78	49	1,7	7,3	14,7	22	29	81	24	59	69	6,5	321	58	99,8	138	3,7	97
123	MS	female	59	73	125	70	88,3	yes	yes	yes	no	16,8	1,22	5,44	77	45	5,8	9,6	6,3	22	21	65	17	128	159	6,8	321	95	57,0	139	4,6	98
124	MS	female	58	59	135	80	98,3	yes	yes	no	no	13,1	1,38	7,71	75	49	0,9	2,4	6,0	20	23	85	17	226	157	6,1	280	81	69,8	141	4,9	102
125	Healthy	male	57	64	120	75	90,0	no	no	yes		29,5	2,70	5,38	72	49	2,6	5,3	14,0	18	18	60	49	63	134	8,5	309	112	62,3	139	4,6	101
126	MS	female	58	74	135	80	98,3	yes	yes	no	no	8,5	1,27	5,33	79	49	0,5	6,1	17,1	26	14	86	19	312	175	8,0	339	73	79,1	140	4,4	100
127	Healthy	male	61	72	120	80	93,3	no	no	no		13,7	1,48	4,94	88	52	1,5	4,0	17,1	107	143	57	62	657	241	5,1	268	96	72,9	139	5	99
128	Healthy	male	62	51	105	60	75,0	no	no	yes		21,4	1,85	5,11	67	46	0,5	6,8	16,1	23	20	64	15	73	152	3,0	345	77	92,5	142	4,2	104
129	Healthy	male	56	76	130	80	96,7	no	no	yes		7,8	1,09	4,33	77	51	8,5	8,2	9,1	20	23	86	32	83	147	6,6	399	95	77,2	140	4,1	103
130	Healthy	male	56	66	130	85	100,0	no	no	yes		12,5	1,74	5,55	75	51	0,5	1,3	13,7	23	20	47	32	115	185	6,3	339	106	67,2	141	4,9	102
