# Supplementary material for: Sex-Related Differences in the Associations between Adiponectin and Serum Lipoproteins in Healthy Subjects and Patients with Metabolic Syndrome
Source: Biomedicines. 2024 Sep 1;12(9):1972. doi: 10.3390/biomedicines12091972 (PMC11429094; doi:10.3390/biomedicines12091972)
Supplement: Supplementary file 1 [file biomedicines-12-01972-s001.zip › Table S10.pdf]

Table S10. Correlation analyses of CRP with serum levels of VLDL, IDL, LDL, and HDL, performed separately in healthy females and males, as well as females and males with MS.

	CRP (μg/mL)							
	Healthy				MS			
	Female (N=31)		Male (N=34)		Female (N=31)		Male (N=34)	
**Variable (mg/dL)**	**r**	**p**	**r**	**p**	**r**	**p**	**r**	**p**
**VLDL**								
VLDL1-C	0.05	0.8005	0.09	0.6315	-0.04	0.8398	-0.17	0.3395
VLDL2-C	0.13	0.4913	0.16	0.3558	0.07	0.6917	-0.20	0.2681
VLDL3-C	0.15	0.4301	0.19	0.2731	0.13	0.4948	-0.19	0.2892
VLDL4-C	-0.02	0.9337	0.34	0.0525	0.10	0.5824	-0.18	0.3110
VLDL5-C	-0.27	0.1418	0.27	0.1171	0.00	0.9979	-0.10	0.5881
VLDL1-FC	0.10	0.5997	0.13	0.4653	-0.04	0.8268	-0.19	0.2698
VLDL2-FC	0.12	0.5355	0.13	0.4576	0.08	0.6694	-0.16	0.3580
VLDL3-FC	0.10	0.6012	0.14	0.4246	0.09	0.6456	-0.20	0.2553
VLDL4-FC	0.01	0.9742	0.34	0.0498	0.04	0.8415	-0.17	0.3298
VLDL5-FC	-0.41	0.0232	0.34	0.0508	0.03	0.8779	0.03	0.8549
VLDL1-TG	0.04	0.8122	-0.02	0.8901	-0.06	0.7528	-0.19	0.2925
VLDL2-TG	0.14	0.4554	0.10	0.5609	0.03	0.8572	-0.19	0.2758
VLDL3-TG	0.09	0.6388	0.13	0.4645	0.05	0.7958	-0.16	0.3674
VLDL4-TG	-0.04	0.8206	0.20	0.2629	0.00	0.9802	-0.11	0.5492
VLDL5-TG	-0.33	0.0671	0.23	0.1885	-0.03	0.8920	0.00	0.9815
VLDL1-PL	0.10	0.6111	0.01	0.9507	0.02	0.9022	-0.14	0.4144
VLDL2-PL	0.15	0.4206	0.13	0.4517	0.10	0.6072	-0.19	0.2908
VLDL3-PL	0.10	0.6020	0.14	0.4241	0.11	0.5584	-0.16	0.3561
VLDL4-PL	0.00	0.9931	0.32	0.0616	0.05	0.7979	-0.13	0.4528
VLDL5-PL	-0.21	0.2664	0.26	0.1375	0.02	0.9039	-0.02	0.9149
VLDL-apoB	0.03	0.8892	0.14	0.4409	0.03	0.8937	-0.15	0.3988
**IDL**								

	CRP (μg/mL)							
	Healthy				MS			
	Female (N=31)		Male (N=34)		Female (N=31)		Male (N=34)	
**Variable (mg/dL)**	**r**	**p**	**r**	**p**	**r**	**p**	**r**	**p**
IDL-C	-0.01	0.9707	0.43	0.0111	0.11	0.5690	-0.14	0.4465
IDL-FC	-0.01	0.9423	0.41	0.0167	0.05	0.7992	-0.14	0.4290
IDL-TG	-0.03	0.8807	0.08	0.6519	-0.05	0.7950	-0.19	0.2880
IDL-PL	-0.02	0.9012	0.23	0.1828	-0.08	0.6644	-0.18	0.3102
IDL-apoB	-0.06	0.7640	0.39	0.0216	0.09	0.6148	-0.08	0.6655
**LDL**								
LDL1-C	-0.21	0.2493	0.36	0.0391	-0.10	0.5749	0.01	0.9617
LDL2-C	-0.18	0.3417	0.15	0.3968	-0.02	0.9116	0.34	0.0498
LDL3-C	-0.06	0.7557	0.17	0.3239	-0.13	0.5017	0.11	0.5297
LDL4-C	0.03	0.8884	0.24	0.1686	-0.13	0.4784	-0.04	0.8314
LDL5-C	0.03	0.8841	0.08	0.6569	-0.07	0.7009	-0.08	0.6394
LDL6-C	0.12	0.5171	-0.10	0.5814	0.02	0.9236	-0.03	0.8731
LDL1-FC	-0.18	0.3235	0.33	0.0560	-0.11	0.5392	0.02	0.8962
LDL2-FC	-0.19	0.3006	0.10	0.5849	-0.05	0.7734	0.34	0.0501
LDL3-FC	-0.11	0.5478	0.06	0.7397	-0.15	0.4226	0.09	0.6125
LDL4-FC	0.02	0.9226	0.11	0.5412	-0.14	0.4570	-0.03	0.8758
LDL5-FC	0.01	0.9664	0.06	0.7202	-0.06	0.7578	-0.11	0.5371
LDL6-FC	0.14	0.4564	-0.10	0.5889	0.02	0.9214	-0.07	0.6968
LDL1-TG	-0.06	0.7631	0.30	0.0860	0.14	0.4478	0.03	0.8508
LDL2-TG	-0.15	0.4141	0.34	0.0517	0.13	0.4906	0.24	0.1685
LDL3-TG	-0.31	0.0903	0.29	0.0951	-0.01	0.9639	0.27	0.1203
LDL4-TG	0.02	0.9265	0.32	0.0644	0.16	0.3885	0.07	0.6781
LDL5-TG	0.00	0.9888	0.09	0.6098	0.21	0.2479	0.00	0.9897
LDL6-TG	0.21	0.2609	-0.22	0.2110	0.25	0.1693	0.06	0.7545
LDL1-PL	-0.21	0.2523	0.40	0.0193	-0.06	0.7357	0.03	0.8803
LDL2-PL	-0.15	0.4068	0.18	0.3141	-0.03	0.8563	0.35	0.0409
LDL3-PL	-0.06	0.7681	0.18	0.3061	-0.09	0.6286	0.13	0.4486

	CRP (μg/mL)							
	Healthy				MS			
	Female (N=31)		Male (N=34)		Female (N=31)		Male (N=34)	
**Variable (mg/dL)**	**r**	**p**	**r**	**p**	**r**	**p**	**r**	**p**
LDL4-PL	0.03	0.8765	0.26	0.1354	-0.14	0.4683	-0.04	0.8013
LDL5-PL	0.03	0.8799	0.08	0.6631	-0.02	0.9116	-0.09	0.6149
LDL6-PL	0.08	0.6576	-0.11	0.5248	0.01	0.9390	-0.05	0.7941
LDL1-apoB	-0.22	0.2425	0.40	0.0189	-0.06	0.7357	0.05	0.7696
LDL2-apoB	-0.18	0.3394	0.18	0.2971	0.00	0.9862	0.40	0.0204
LDL3-apoB	-0.06	0.7511	0.20	0.2558	-0.06	0.7300	0.13	0.4688
LDL4-apoB	0.04	0.8510	0.28	0.1036	-0.10	0.5750	-0.05	0.7676
LDL5-apoB	0.03	0.8765	0.08	0.6669	0.03	0.8809	-0.05	0.7696
LDL6-apoB	0.08	0.6591	-0.06	0.7553	0.07	0.7122	-0.01	0.9412
**HDL**								
HDL1-C	-0.15	0.4263	-0.16	0.3715	-0.04	0.8142	0.04	0.8054
HDL2-C	-0.18	0.3389	0.11	0.5374	0.06	0.7422	0.02	0.9302
HDL3-C	-0.18	0.3317	0.13	0.4672	-0.14	0.4612	-0.08	0.6456
HDL4-C	0.00	0.9966	-0.23	0.2002	-0.45	0.0103	-0.12	0.5020
HDL1-FC	-0.14	0.4457	-0.12	0.4918	-0.17	0.3539	-0.09	0.6142
HDL2-FC	-0.12	0.5032	0.18	0.3097	-0.10	0.5929	-0.04	0.8277
HDL3-FC	-0.14	0.4383	0.09	0.6044	-0.16	0.3839	-0.13	0.4470
HDL4-FC	-0.03	0.8701	-0.07	0.6781	-0.29	0.1096	-0.16	0.3632
HDL1-TG	-0.13	0.4892	-0.03	0.8496	0.21	0.2589	-0.22	0.2174
HDL2-TG	-0.07	0.7056	0.24	0.1695	0.27	0.1376	-0.11	0.5359
HDL3-TG	-0.02	0.9016	0.25	0.1530	0.24	0.1946	-0.08	0.6537
HDL4-TG	0.01	0.9587	0.01	0.9408	-0.03	0.8852	-0.25	0.1480
HDL1-PL	-0.19	0.2981	-0.04	0.8234	0.06	0.7446	0.00	0.9986
HDL2-PL	-0.19	0.3043	0.23	0.1826	0.05	0.7800	0.05	0.7624
HDL3-PL	-0.18	0.3444	0.19	0.2740	-0.12	0.5334	-0.07	0.6844
HDL4-PL	0.05	0.8013	-0.03	0.8556	-0.32	0.0834	-0.13	0.4688

Table S10

	CRP (µg/mL)							
	**Healthy**				**MS**			
	**Female (N=31)**		**Male (N=34)**		**Female (N=31)**		**Male (N=34)**	
**Variable (mg/dL)**	**r**	**p**	**r**	**p**	**r**	**p**	**r**	**p**
HDL1-apoA-I	-0.18	0.3322	-0.12	0.4860	0.03	0.8741	-0.07	0.6869
HDL2-apoA-I	-0.20	0.2764	-0.01	0.9439	-0.02	0.9090	0.00	0.9976
HDL3-apoA-I	-0.15	0.4352	0.16	0.3796	-0.17	0.3527	-0.05	0.7987
HDL4-apoA-I	0.05	0.8097	-0.19	0.2890	-0.41	0.0237	-0.25	0.1508
HDL1-apoA-II	-0.21	0.2632	-0.09	0.6326	0.07	0.7065	-0.11	0.5328
HDL2-apoA-II	-0.24	0.1961	0.20	0.2502	0.06	0.7463	-0.02	0.9159
HDL3-apoA-II	-0.16	0.3811	0.29	0.0992	-0.13	0.4825	-0.10	0.5707
HDL4-apoA-II	0.01	0.9638	-0.12	0.5091	-0.49	0.0056	-0.25	0.1619

Spearman correlation analyses were used to evaluate associations of CRP with the serum levels of VLDL, IDL, LDL, and HDL. Spearman correlation coefficients with $|r| \geq 0.5$ are depicted in bold. ApoA-I, apolipoprotein A-I, apoA-II, apolipoprotein A-II; apoB, apolipoprotein B; C, cholesterol; CRP, C-reactive protein; FC, free cholesterol; HDL, high-density lipoprotein; IDL, intermediate-density lipoprotein; LDL, low-density lipoprotein; MS; metabolic syndrome patient; VLDL, very low-density lipoprotein; PL, phospholipid; TG, triglyceride.

Table S10
