# Supplementary material for: Sex-Related Differences in the Associations between Adiponectin and Serum Lipoproteins in Healthy Subjects and Patients with Metabolic Syndrome
Source: Biomedicines. 2024 Sep 1;12(9):1972. doi: 10.3390/biomedicines12091972 (PMC11429094; doi:10.3390/biomedicines12091972)
Supplement: Supplementary file 1 [file biomedicines-12-01972-s001.zip › Table S2.pdf]

Table S2. Differences in serum levels of VLDL, IDL, LDL, and HDL between healthy females and males, as well as females and males with MS.

	Healthy			MS		
**Variable (mg/dL)**	**Female (N=31)**	**Male (N=34)**	**p-value**	**Female (N=31)**	**Male (N=34)**	**p-value**
**VLDL**						
VLDL1-C	4.53 (3.06, 6.71)	5.59 (3.51, 8.26)	0.2478	7.29 (4.08, 14.00)	10.34 (5.63, 15.96)	0.1181
VLDL2-C	2.22 (1.60, 3.21)	2.46 (1.37, 3.60)	0.9111	2.40 (1.31, 5.72)	3.88 (1.96, 6.59)	0.1276
VLDL3-C	2.78 (1.87, 4.43)	2.92 (2.01, 4.59)	0.9111	4.28 (2.04, 7.18)	4.68 (3.07, 8.09)	0.2320
VLDL4-C	4.95 (3.72, 5.59)	4.29 (2.69, 6.29)	0.7776	5.98 (3.90, 8.70)	6.77 (4.51, 8.76)	0.4423
VLDL5-C	1.35 (0.92, 1.60)	1.02 (0.77, 1.43)	0.1377	1.28 (0.85, 1.72)	1.08 (0.74, 1.53)	0.3510
VLDL1-FC	1.31 (0.73, 2.28)	2.06 (1.26, 3.04)	0.0552	2.50 (1.10, 4.24)	3.81 (2.06, 5.42)	0.1699
VLDL2-FC	0.85 (0.38, 1.46)	0.94 (0.43, 1.52)	0.5857	1.13 (0.42, 2.50)	1.74 (0.88, 2.74)	0.1120
VLDL3-FC	1.06 (0.67, 1.92)	1.19 (0.69, 1.93)	0.8284	1.96 (0.78, 3.10)	2.38 (1.36, 3.50)	0.2269
VLDL4-FC	2.11 (1.44, 2.67)	1.93 (1.17, 2.92)	0.6039	3.06 (1.75, 4.29)	2.85 (2.25, 4.23)	0.6504
VLDL5-FC	0.70 (0.55, 0.98)	0.50 (0.34, 0.70)	0.0088	0.76 (0.45, 1.00)	0.78 (0.56, 1.01)	0.7278
VLDL1-TG	15.45 (9.56, 26.86)	26.88 (19.63, 41.47)	0.0061	43.35 (21.88, 63.02)	48.19 (29.36, 77.43)	0.2587
VLDL2-TG	7.19 (4.54, 11.14)	8.93 (5.26, 12.17)	0.4230	11.65 (7.21, 20.08)	14.45 (9.46, 21.83)	0.1825
VLDL3-TG	7.65 (4.74, 12.27)	7.71 (5.07, 11.64)	0.8233	11.04 (6.36, 18.86)	12.75 (9.56, 19.88)	0.2269
VLDL4-TG	6.82 (5.46, 9.13)	6.96 (4.63, 9.64)	0.9163	10.15 (7.12, 13.62)	10.75 (8.80, 13.49)	0.4580
VLDL5-TG	2.64 (2.25, 2.96)	2.49 (2.09, 2.80)	0.3376	2.93 (2.62, 3.61)	3.05 (2.51, 3.48)	0.8696
VLDL1-PL	2.41 (1.52, 4.75)	4.40 (3.20, 6.56)	0.0219	7.29 (3.47, 9.99)	8.66 (4.58, 12.09)	0.1561
VLDL2-PL	1.95 (1.28, 2.81)	2.37 (1.37, 3.11)	0.3510	2.96 (1.76, 4.93)	3.89 (2.50, 5.60)	0.1504
VLDL3-PL	2.68 (1.68, 3.99)	2.74 (1.80, 4.30)	0.7526	3.95 (2.02, 6.16)	4.74 (3.08, 7.10)	0.2026
VLDL4-PL	3.82 (3.00, 4.68)	3.67 (2.50, 5.17)	0.9738	5.19 (3.62, 6.90)	5.48 (4.22, 6.97)	0.4741
VLDL5-PL	1.57 (1.23, 2.04)	1.42 (1.08, 1.79)	0.2903	1.82 (1.19, 2.13)	1.66 (1.46, 2.12)	0.8644
VLDL-apoB	6.08 (4.31, 8.26)	6.33 (4.08, 9.22)	0.6552	9.20 (5.97, 12.97)	10.39 (6.65, 13.36)	0.3545
**IDL**						
IDL-C	13.62 (10.59, 17.54)	15.80 (10.02, 19.77)	0.6552	15.49 (11.66, 26.31)	19.13 (13.74, 27.15)	0.4620
IDL-FC	3.99 (3.10, 4.98)	4.46 (2.70, 5.70)	0.7476	4.75 (3.18, 7.58)	5.39 (3.85, 7.62)	0.5198
IDL-TG	6.32 (3.96, 9.12)	8.03 (4.72, 12.76)	0.2614	11.53 (6.62, 20.48)	14.68 (9.20, 23.94)	0.2121
IDL-PL	6.91 (6.26, 8.30)	7.78 (6.04, 9.65)	0.5812	7.78 (5.81, 12.71)	9.62 (7.22, 12.47)	0.3150

	Healthy			MS		
Variable (mg/dL)	Female (N=31)	Male (N=34)	p-value	Female (N=31)	Male (N=34)	p-value
IDL-apoB	5.13 (4.12, 6.56)	5.65 (3.81, 6.97)	0.7426	6.43 (5.18, 9.09)	7.06 (5.01, 8.51)	0.8080
**LDL**						
LDL1-C	33.60 (28.65, 38.98)	28.16 (24.98, 33.46)	0.0034	27.10 (22.86, 31.54)	23.27 (20.80, 29.60)	0.1309
LDL2-C	26.32 (23.48, 32.36)	25.92 (16.44, 29.14)	0.1599	19.16 (12.40, 22.58)	16.70 (12.17, 20.07)	0.3443
LDL3-C	23.80 (21.57, 28.68)	23.98 (20.20, 30.27)	0.9581	18.70 (10.26, 22.51)	17.06 (9.85, 21.93)	0.5812
LDL4-C	17.26 (14.37, 21.59)	22.33 (19.41, 26.60)	0.0035	16.51 (11.45, 22.27)	18.98 (11.64, 24.12)	0.7526
LDL5-C	12.74 (9.88, 16.96)	20.22 (15.51, 24.37)	**< 0.0001**	17.25 (13.12, 22.00)	17.64 (12.76, 25.11)	0.6839
LDL6-C	17.06 (13.24, 19.32)	20.81 (18.30, 28.22)	**0.0002**	20.06 (16.57, 26.45)	23.22 (17.72, 31.66)	0.1825
LDL1-FC	10.85 (9.21, 11.98)	8.96 (8.07, 10.43)	0.0024	8.77 (7.64, 10.16)	7.74 (6.95, 9.45)	0.0830
LDL2-FC	8.87 (7.84, 10.66)	8.46 (5.44, 9.30)	0.0784	6.71 (5.29, 7.83)	5.88 (4.88, 7.21)	0.2504
LDL3-FC	7.99 (7.27, 9.46)	7.76 (6.40, 9.12)	0.4154	6.54 (4.42, 7.30)	6.42 (4.30, 7.36)	0.5114
LDL4-FC	5.81 (4.88, 7.03)	6.79 (6.12, 8.03)	0.0126	6.03 (4.38, 6.93)	5.83 (4.73, 7.40)	0.8955
LDL5-FC	4.26 (3.56, 5.61)	6.06 (5.03, 6.97)	**0.0001**	5.34 (4.72, 6.75)	5.30 (4.45, 7.07)	0.9843
LDL6-FC	5.29 (4.48, 5.96)	6.28 (5.50, 7.19)	**0.0002**	6.30 (5.13, 7.23)	6.49 (5.61, 7.94)	0.3443
LDL-TG	19.77 (17.20, 23.99)	20.28 (16.05, 23.53)	0.3477	24.34 (20.59, 28.91)	23.71 (20.12, 28.04)	0.6936
LDL1-TG	6.78 (5.96, 8.30)	5.45 (4.60, 6.75)	0.0024	8.16 (6.60, 8.57)	6.99 (6.13, 8.18)	0.1091
LDL2-TG	2.93 (2.64, 3.62)	2.41 (1.83, 2.77)	**0.0004**	2.89 (2.35, 3.23)	2.49 (2.04, 2.87)	0.1658
LDL3-TG	2.66 (2.42, 3.11)	2.38 (1.93, 2.96)	0.1062	2.61 (2.04, 3.14)	2.20 (1.76, 2.76)	0.1658
LDL4-TG	2.17 (1.68, 2.88)	2.52 (1.59, 3.04)	0.7476	2.90 (2.60, 3.62)	3.08 (1.98, 3.90)	0.6647
LDL5-TG	1.91 (1.46, 2.59)	2.44 (1.43, 3.35)	0.0953	2.67 (2.19, 3.89)	3.42 (2.19, 4.41)	0.4782
LDL6-TG	2.61 (2.12, 3.00)	3.12 (2.60, 3.93)	0.0056	3.77 (2.83, 4.42)	4.03 (2.96, 4.79)	0.1868
LDL-PL	75.03 (67.91, 83.04)	79.27 (67.40, 85.78)	0.4782	66.60 (54.78, 81.72)	65.33 (49.12, 80.10)	0.6552
LDL1-PL	18.75 (15.83, 21.37)	15.32 (13.53, 18.35)	0.0013	15.24 (13.46, 17.59)	13.46 (11.92, 16.06)	0.0773
LDL2-PL	14.37 (13.02, 17.33)	13.88 (8.93, 15.55)	0.1020	10.75 (7.19, 12.51)	9.10 (7.05, 11.05)	0.2874
LDL3-PL	13.16 (11.88, 15.32)	12.75 (10.91, 16.00)	0.7626	10.70 (6.49, 12.51)	9.88 (6.38, 12.02)	0.4701
LDL4-PL	9.33 (8.10, 11.86)	11.98 (10.25, 14.05)	0.0044	9.38 (6.72, 12.36)	10.48 (6.96, 13.02)	0.7726
LDL5-PL	7.33 (5.62, 9.36)	10.98 (8.45, 13.02)	**< 0.0001**	9.59 (7.56, 11.89)	9.73 (7.36, 13.78)	0.7180
LDL6-PL	10.36 (8.82, 11.32)	12.05 (10.52, 15.16)	**0.0002**	12.23 (10.07, 14.41)	13.16 (10.75, 16.91)	0.1804
LDL1-apoB	17.31 (14.86, 20.16)	14.32 (12.63, 17.13)	0.0010	14.81 (12.78, 17.02)	12.87 (11.17, 15.71)	0.0679

	Healthy			MS		
Variable (mg/dL)	Female (N=31)	Male (N=34)	p-value	Female (N=31)	Male (N=34)	p-value
LDL2-apoB	14.43 (12.88, 16.86)	13.57 (9.41, 15.50)	0.0953	10.72 (7.22, 13.27)	9.65 (7.61, 11.45)	0.3214
LDL3-apoB	13.72 (12.43, 16.24)	13.60 (11.39, 16.68)	0.6363	11.44 (7.24, 14.16)	10.49 (6.77, 12.95)	0.3278
LDL4-apoB	10.48 (9.22, 13.93)	14.16 (11.49, 17.02)	0.0081	11.67 (8.77, 15.67)	12.51 (8.29, 16.18)	0.8903
LDL5-apoB	9.10 (6.68, 12.14)	13.87 (10.37, 17.65)	**0.0002**	12.14 (10.11, 16.44)	12.78 (9.76, 18.35)	0.7576
LDL6-apoB	13.72 (11.16, 16.10)	16.71 (14.40, 22.70)	0.0005	16.37 (13.59, 22.66)	19.79 (14.66, 26.95)	0.1804
**HDL**						
HDL1-C	23.61 (16.16, 31.43)	17.56 (14.34, 20.85)	0.0083	16.70 (14.22, 21.13)	14.16 (11.75, 18.02)	0.0269
HDL2-C	9.61 (8.62, 13.08)	8.79 (7.77, 10.14)	0.0762	8.20 (7.57, 9.46)	8.12 (6.48, 9.73)	0.1935
HDL3-C	12.44 (10.79, 13.70)	11.94 (10.69, 13.41)	0.5241	10.92 (9.78, 11.99)	10.34 (9.31, 11.83)	0.3056
HDL4-C	21.28 (17.21, 23.44)	23.20 (20.39, 25.04)	0.0080	19.22 (16.24, 21.91)	18.73 (15.97, 23.14)	0.9895
HDL1-FC	6.72 (4.98, 8.15)	5.08 (4.63, 5.94)	0.0256	4.68 (3.83, 5.41)	4.28 (3.62, 5.05)	0.1868
HDL2-FC	2.83 (2.38, 3.18)	2.56 (2.16, 2.88)	0.0865	2.35 (2.09, 2.74)	2.18 (1.95, 2.68)	0.2586
HDL3-FC	2.84 (2.62, 3.42)	3.03 (2.56, 3.34)	0.9948	2.61 (2.21, 2.95)	2.45 (2.07, 3.07)	0.4620
HDL4-FC	4.44 (3.68, 5.46)	5.06 (4.40, 5.66)	0.0853	4.55 (3.80, 5.01)	4.24 (3.75, 5.24)	0.8284
HDL1-TG	3.89 (3.20, 5.25)	2.62 (2.00, 3.01)	**< 0.0001**	3.75 (3.33, 5.59)	2.90 (2.25, 3.81)	0.0235
HDL2-TG	2.02 (1.56, 2.46)	1.50 (1.20, 1.78)	**0.0003**	2.21 (1.77, 2.75)	1.94 (1.56, 2.38)	0.1413
HDL3-TG	2.29 (1.90, 2.83)	1.99 (1.61, 2.20)	0.0089	2.68 (2.16, 3.04)	2.68 (1.99, 3.13)	0.6316
HDL4-TG	3.15 (2.40, 4.10)	3.45 (2.61, 3.75)	0.8182	3.67 (3.25, 4.62)	3.89 (3.41, 4.71)	0.5678
HDL1-PL	29.68 (19.85, 37.80)	19.77 (16.92, 23.90)	0.0029	20.57 (17.18, 26.74)	17.93 (14.24, 20.84)	0.0184
HDL2-PL	15.68 (13.20, 18.82)	13.69 (11.81, 15.09)	0.0187	13.78 (11.22, 15.62)	13.04 (9.98, 15.06)	0.1935
HDL3-PL	19.77 (17.86, 21.01)	18.53 (16.27, 20.95)	0.2424	17.82 (15.54, 19.55)	16.48 (15.00, 19.30)	0.2121
HDL4-PL	29.18 (25.48, 31.46)	30.45 (27.79, 33.31)	0.0496	26.03 (24.25, 30.45)	26.86 (22.72, 30.96)	0.9163
HDL1-apoA-I	38.36 (26.12, 52.62)	25.32 (19.54, 31.49)	0.0022	25.82 (21.50, 33.16)	22.45 (17.70, 27.43)	0.0328
HDL2-apoA-I	22.07 (18.31, 24.05)	18.73 (16.52, 21.44)	0.0174	18.06 (15.85, 20.31)	17.33 (14.82, 21.02)	0.3377
HDL3-apoA-I	32.18 (28.34, 33.61)	29.82 (27.12, 33.38)	0.2532	30.26 (25.38, 31.70)	28.21 (24.55, 32.99)	0.6363
HDL4-apoA-I	77.59 (68.67, 87.97)	85.90 (78.56, 92.48)	0.0239	75.75 (68.62, 84.68)	75.18 (68.64, 90.02)	0.9529
HDL1-apoA-II	3.35 (2.15, 4.80)	2.38 (2.04, 2.81)	0.0162	2.10 (1.56, 3.48)	2.04 (1.74, 2.70)	0.6316
HDL2-apoA-II	4.20 (3.77, 4.69)	3.92 (3.30, 4.33)	0.0612	3.76 (2.75, 4.72)	3.64 (3.10, 4.20)	0.9633
HDL3-apoA-II	7.31 (6.90, 8.07)	7.37 (6.86, 8.08)	0.9476	7.18 (6.10, 8.28)	7.09 (6.26, 8.73)	0.7327

Table S2

	Healthy			MS		
**Variable (mg/dL)**	**Female (N=31)**	**Male (N=34)**	**p-value**	**Female (N=31)**	**Male (N=34)**	**p-value**
HDL4-apoA-II	18.68 (16.24, 22.42)	22.11 (19.20, 24.32)	0.0025	18.67 (16.61, 21.32)	19.49 (17.41, 22.73)	0.3214

Data are presented as median (q1, q3). Differences between the groups were tested using the Mann-Whitney U test. *p*-values < 0.0005 are considered statistically significant after a Bonferroni correction for multiple testing and are depicted in bold. ApoA-I, apolipoprotein A-I; apoA-II, apolipoprotein A-II; apoB, apolipoprotein B; C, cholesterol; FC, free cholesterol; HDL, high-density lipoprotein; IDL, intermediate-density lipoprotein; LDL, low-density lipoprotein; MS; metabolic syndrome patient; VLDL, very low-density lipoprotein; PL, phospholipid; TG, triglyceride.

Table S2
