# Supplementary material for: Sex-Related Differences in the Associations between Adiponectin and Serum Lipoproteins in Healthy Subjects and Patients with Metabolic Syndrome
Source: Biomedicines. 2024 Sep 1;12(9):1972. doi: 10.3390/biomedicines12091972 (PMC11429094; doi:10.3390/biomedicines12091972)
Supplement: Supplementary file 1 [file biomedicines-12-01972-s001.zip › Table S3.pdf]

Table S3. Correlation analyses of serum levels of adiponectin with serum levels of VLDL, IDL, LDL, and HDL, performed separately in healthy females and males, as well as females and males with MS.

	Healthy				MS			
	Female (N=31)		Male (N=34)		Female (N=31)		Male (N=34)	
Variable (mg/dL)	r	p	r	p	r	p	r	p
**VLDL**								
VLDL1-C	-0.27	0.1363	**-0.51**	0.0021	-0.13	0.4961	-0.12	0.4987
VLDL2-C	-0.36	0.0454	**-0.50**	0.0025	-0.18	0.3307	-0.08	0.6666
VLDL3-C	-0.42	0.0193	**-0.54**	0.0009	-0.22	0.2420	-0.06	0.7326
VLDL4-C	-0.30	0.1066	**-0.54**	0.0010	-0.17	0.3526	0.04	0.8415
VLDL5-C	0.05	0.7851	-0.13	0.4556	0.03	0.8729	0.31	0.0719
VLDL1-FC	-0.30	0.1008	**-0.54**	0.0009	-0.20	0.2728	-0.18	0.3154
VLDL2-FC	-0.38	0.0327	-0.48	0.0041	-0.23	0.2172	-0.12	0.4965
VLDL3-FC	-0.38	0.0342	**-0.53**	0.0014	-0.20	0.2782	-0.11	0.5351
VLDL4-FC	-0.35	0.0547	**-0.53**	0.0013	-0.18	0.3296	0.00	0.9863
VLDL5-FC	0.04	0.8185	-0.38	0.0285	0.05	0.7810	0.01	0.9467
VLDL1-TG	-0.27	0.1428	**-0.56**	0.0006	-0.24	0.2026	-0.28	0.1129
VLDL2-TG	-0.37	0.0383	**-0.53**	0.0011	-0.20	0.2821	-0.15	0.3940
VLDL3-TG	-0.39	0.0280	**-0.52**	0.0016	-0.22	0.2375	-0.13	0.4529
VLDL4-TG	-0.32	0.0835	**-0.58**	0.0004	-0.25	0.1682	-0.10	0.5881
VLDL5-TG	0.12	0.5034	-0.16	0.3738	0.02	0.9108	0.09	0.6278
VLDL1-PL	-0.31	0.0915	**-0.59**	0.0002	-0.21	0.2651	-0.27	0.1288
VLDL2-PL	-0.40	0.0242	**-0.51**	0.0020	-0.24	0.1874	-0.15	0.3875
VLDL3-PL	-0.40	0.0241	**-0.53**	0.0014	-0.24	0.1982	-0.13	0.4509
VLDL4-PL	-0.35	0.0509	**-0.56**	0.0005	-0.24	0.2013	-0.04	0.8295
VLDL5-PL	-0.06	0.7338	-0.27	0.1157	-0.02	0.9142	0.19	0.2832
VLDL-apoB	-0.34	0.0638	**-0.55**	0.0008	-0.21	0.2524	-0.10	0.5869
**IDL**								
IDL-C	-0.33	0.0692	**-0.54**	0.0011	-0.09	0.6197	-0.02	0.9153

	**Healthy**				**MS**			
	**Female (N=31)**		**Male (N=34)**		**Female (N=31)**		**Male (N=34)**	
**Variable (mg/dL)**	**r**	**p**	**r**	**p**	**r**	**p**	**r**	**p**
IDL-FC	-0.36	0.0479	**-0.54**	0.0009	-0.12	0.5274	-0.04	0.8382
IDL-TG	-0.23	0.2151	**-0.52**	0.0015	-0.09	0.6474	-0.15	0.3921
IDL-PL	-0.34	0.0633	-0.49	0.0034	-0.02	0.9194	-0.09	0.6163
IDL-apoB	-0.24	0.1974	**-0.55**	0.0007	-0.12	0.5345	-0.05	0.7995
**LDL**								
LDL1-C	0.03	0.8802	-0.03	0.8536	0.47	0.0073	0.29	0.0920
LDL2-C	0.25	0.1752	0.21	0.2445	0.38	0.0347	-0.10	0.5745
LDL3-C	-0.01	0.9596	0.10	0.5720	0.43	0.0166	0.11	0.5299
LDL4-C	-0.32	0.0749	-0.35	0.0416	0.27	0.1397	0.16	0.3655
LDL5-C	-0.23	0.2155	**-0.61**	0.0001	0.00	0.9837	-0.03	0.8854
LDL6-C	-0.01	0.9764	-0.39	0.0244	-0.12	0.5252	-0.16	0.3670
LDL1-FC	0.01	0.9596	-0.04	0.8348	0.46	0.0092	0.29	0.0959
LDL2-FC	0.27	0.1437	0.22	0.2078	0.43	0.0154	-0.09	0.6236
LDL3-FC	0.09	0.6427	0.16	0.3785	**0.51**	0.0036	0.14	0.4252
LDL4-FC	-0.22	0.2261	-0.25	0.1599	0.33	0.0718	0.21	0.2363
LDL5-FC	-0.14	0.4680	**-0.57**	0.0005	0.06	0.7343	0.05	0.7882
LDL6-FC	0.15	0.4147	-0.36	0.0372	-0.07	0.6924	-0.15	0.3998
LDL1-TG	-0.11	0.5642	-0.34	0.0457	0.05	0.8043	0.11	0.5527
LDL2-TG	-0.08	0.6594	-0.09	0.6065	0.20	0.2916	0.03	0.8800
LDL3-TG	0.22	0.2407	-0.08	0.6333	0.29	0.1100	0.07	0.7004
LDL4-TG	-0.37	0.0384	-0.43	0.0102	0.01	0.9476	0.10	0.5842
LDL5-TG	-0.28	0.1225	**-0.51**	0.0020	-0.17	0.3710	-0.05	0.7763
LDL6-TG	0.20	0.2860	-0.19	0.2704	-0.19	0.3102	-0.15	0.3858
LDL1-PL	0.04	0.8429	-0.05	0.7651	0.50	0.0043	0.31	0.0783
LDL2-PL	0.25	0.1756	0.22	0.2029	0.41	0.0208	-0.04	0.8436
LDL3-PL	-0.02	0.9125	0.11	0.5196	0.44	0.0133	0.12	0.4855
LDL4-PL	-0.36	0.0495	-0.37	0.0310	0.29	0.1152	0.17	0.3481
LDL5-PL	-0.23	0.2110	**-0.59**	0.0002	0.00	0.9828	0.00	0.9932

	Healthy				MS			
	Female (N=31)		Male (N=34)		Female (N=31)		Male (N=34)	
**Variable (mg/dL)**	**r**	**p**	**r**	**p**	**r**	**p**	**r**	**p**
LDL6-PL	0.13	0.4979	-0.32	0.0637	-0.09	0.6405	-0.17	0.3340
LDL1-apoB	0.07	0.6963	-0.05	0.7974	0.47	0.0075	0.29	0.0915
LDL2-apoB	0.21	0.2598	0.18	0.3023	0.40	0.0263	-0.11	0.5187
LDL3-apoB	-0.09	0.6458	0.06	0.7380	0.42	0.0187	0.09	0.6036
LDL4-apoB	-0.39	0.0285	-0.43	0.0114	0.21	0.2524	0.15	0.3857
LDL5-apoB	-0.22	0.2244	**-0.61**	0.0001	-0.06	0.7457	-0.08	0.6682
LDL6-apoB	-0.02	0.9202	-0.42	0.0127	-0.14	0.4561	-0.14	0.4262
**HDL**								
HDL1-C	0.33	0.0697	**0.62**	0.0001	0.39	0.0307	0.01	0.9596
HDL2-C	0.43	0.0147	**0.59**	0.0002	**0.58**	0.0006	0.18	0.3002
HDL3-C	0.32	0.0763	0.45	0.0079	**0.56**	0.0011	0.18	0.3210
HDL4-C	0.09	0.6443	-0.09	0.5939	0.16	0.3779	0.13	0.4483
HDL1-FC	0.30	0.1059	**0.56**	0.0006	**0.51**	0.0036	0.05	0.7641
HDL2-FC	0.32	0.0818	0.47	0.0050	**0.58**	0.0006	0.05	0.7763
HDL3-FC	0.23	0.2166	0.21	0.2369	**0.60**	0.0004	0.20	0.2679
HDL4-FC	0.00	0.9828	-0.13	0.4504	0.36	0.0490	0.21	0.2281
HDL1-TG	0.05	0.7951	0.26	0.1318	0.19	0.3094	0.18	0.2961
HDL2-TG	-0.09	0.6284	-0.04	0.8397	0.17	0.3560	0.13	0.4629
HDL3-TG	-0.29	0.1095	-0.33	0.0528	0.16	0.3847	0.04	0.8255
HDL4-TG	-0.40	0.0266	**-0.57**	0.0004	-0.05	0.7703	0.09	0.5964
HDL1-PL	0.34	0.0635	**0.65**	<0.0001	0.49	0.0056	0.09	0.5976
HDL2-PL	0.42	0.0194	**0.61**	0.0002	**0.57**	0.0007	0.17	0.3227
HDL3-PL	0.38	0.0349	0.42	0.0141	**0.57**	0.0009	0.15	0.3863
HDL4-PL	0.07	0.7164	-0.08	0.6360	0.29	0.1110	0.21	0.2253
HDL1-apoA-I	0.30	0.1051	**0.62**	0.0001	0.42	0.0180	0.06	0.7410
HDL2-apoA-I	0.43	0.0163	0.48	0.0045	**0.53**	0.0020	0.09	0.5979
HDL3-apoA-I	0.25	0.1785	0.41	0.0163	**0.52**	0.0026	0.18	0.3021

	Healthy				MS			
	Female (N=31)		Male (N=34)		Female (N=31)		Male (N=34)	
**Variable (mg/dL)**	**r**	**p**	**r**	**p**	**r**	**p**	**r**	**p**
HDL4-apoA-I	-0.01	0.9631	-0.23	0.1840	0.18	0.3391	0.30	0.0859
HDL1-apoA-II	0.20	0.2792	**0.50**	0.0026	0.43	0.0150	-0.06	0.7358
HDL2-apoA-II	0.28	0.1220	0.29	0.0977	0.42	0.0191	-0.07	0.6896
HDL3-apoA-II	-0.08	0.6529	-0.07	0.6876	0.44	0.0143	-0.05	0.7846
HDL4-apoA-II	-0.10	0.6063	-0.41	0.0154	0.13	0.4747	0.03	0.8719

Spearman correlation analyses were used to evaluate associations of the serum levels of adiponectin with the serum levels of VLDL, IDL, LDL, and HDL. Spearman correlation coefficients with|r|≥0.5 are depicted in bold. ApoA-I, apolipoprotein A-I; apoA-II, apolipoprotein A-II; apoB, apolipoprotein B; C, cholesterol; FC, free cholesterol; HDL, high-density lipoprotein; HV, healthy volunteer; IDL, intermediate-density lipoprotein; LDL, low-density lipoprotein; MS; metabolic syndrome patient; VLDL, very low-density lipoprotein; PL, phospholipid; TG, triglyceride.

Table S3
