# Supplementary material for: Sex-Related Differences in the Associations between Adiponectin and Serum Lipoproteins in Healthy Subjects and Patients with Metabolic Syndrome
Source: Biomedicines. 2024 Sep 1;12(9):1972. doi: 10.3390/biomedicines12091972 (PMC11429094; doi:10.3390/biomedicines12091972)
Supplement: Supplementary file 1 [file biomedicines-12-01972-s001.zip › Table S4.pdf]

Table S4. Partial correlation analyses of serum levels of adiponectin with selected lipoprotein parameters in healthy males and females with MS.

	Adiponectin (μg/mL)									
	Healthy males (N=34)									
	Unadjusted		Model 1		Model 2		Model 3		Model 4	
**Variable (mg/dL)**	**r**	**p**	**r**	**p**	**r**	**p**	**r**	**p**	**r**	**p**
**VLDL**										
VLDL1-C	**-0.51**	0.0021	-0.36	0.0433	-0.33	0.0645	-0.34	0.0573	-0.38	0.0329
VLDL2-C	**-0.50**	0.0025	-0.33	0.0611	-0.30	0.1003	-0.31	0.0822	-0.35	0.0470
VLDL3-C	**-0.54**	0.0009	-0.40	0.0216	-0.37	0.0372	-0.39	0.0286	-0.42	0.0164
VLDL4-C	**-0.54**	0.0010	-0.40	0.0228	-0.37	0.0398	-0.40	0.0228	-0.41	0.0191
VLDL1-FC	**-0.54**	0.0009	-0.42	0.0179	-0.38	0.0321	-0.39	0.0266	-0.44	0.0128
VLDL3-FC	**-0.53**	0.0014	-0.37	0.0352	-0.35	0.0528	-0.36	0.0437	-0.39	0.0282
VLDL4-FC	**-0.53**	0.0013	-0.40	0.0239	-0.36	0.0408	-0.40	0.0232	-0.41	0.0205
VLDL1-TG	**-0.56**	0.0006	-0.41	0.0185	-0.40	0.0229	-0.41	0.0209	-0.43	0.0139
VLDL2-TG	**-0.53**	0.0011	-0.40	0.0237	-0.37	0.0393	-0.38	0.0333	-0.41	0.0182
VLDL3-TG	**-0.52**	0.0016	-0.37	0.0355	-0.34	0.0569	-0.35	0.0475	-0.38	0.0318
VLDL4-TG	**-0.58**	0.0004	-0.46	0.0074	-0.45	0.0099	-0.47	0.0072	-0.48	0.0056
VLDL1-PL	**-0.59**	0.0002	-0.46	0.0082	-0.42	0.0162	-0.43	0.0142	-0.46	0.0076
VLDL2-PL	**-0.51**	0.0020	-0.38	0.0334	-0.33	0.0610	-0.35	0.0508	-0.39	0.0254
VLDL3-PL	**-0.53**	0.0014	-0.39	0.0279	-0.36	0.0406	-0.38	0.0335	-0.41	0.0211
VLDL4-PL	**-0.56**	0.0005	-0.44	0.0112	-0.41	0.0208	-0.44	0.0114	-0.45	0.0089
VLDL-apoB	**-0.55**	0.0008	-0.41	0.0205	-0.39	0.0283	-0.40	0.0233	-0.42	0.0159
**IDL**										
IDL-C	**-0.54**	0.0011	-0.48	0.0054	-0.43	0.0139	-0.48	0.0050	-0.47	0.0062
IDL-FC	**-0.54**	0.0009	-0.48	0.0051	-0.44	0.0127	-0.48	0.0052	-0.48	0.0052
IDL-TG	**-0.52**	0.0015	-0.40	0.0222	-0.38	0.0312	-0.39	0.0273	-0.41	0.0190
IDL-apoB	**-0.55**	0.0007	-0.47	0.0073	-0.45	0.0106	-0.49	0.0041	-0.47	0.0070

**LDL**										
LDL5-C	**-0.61**	0.0001	**-0.56**	0.0010	**-0.55**	0.0012	**-0.55**	0.0010	**-0.55**	0.0011
LDL5-FC	**-0.57**	0.0005	**-0.53**	0.0020	**-0.52**	0.0024	**-0.52**	0.0020	**-0.52**	0.0023
LDL5-TG	**-0.51**	0.0020	-0.38	0.0308	-0.38	0.0344	-0.39	0.0294	-0.41	0.0205
LDL5-PL	**-0.59**	0.0002	**-0.54**	0.0015	**-0.53**	0.0020	**-0.53**	0.0017	**-0.53**	0.0017
LDL5-apoB	**-0.61**	0.0001	**-0.54**	0.0014	**-0.53**	0.0018	**-0.54**	0.0015	**-0.54**	0.0015
**HDL**										
HDL1-C	**0.62**	0.0001	0.48	0.0058	0.47	0.0071	0.48	0.0056	0.47	0.0065
HDL2-C	**0.59**	0.0002	0.40	0.0246	0.38	0.0325	0.39	0.0261	0.39	0.0253
HDL1-FC	**0.56**	0.0006	0.41	0.0207	0.39	0.0295	0.40	0.0246	0.40	0.0237
HDL4-TG	**-0.57**	0.0004	-0.42	0.0159	-0.44	0.0125	-0.44	0.0118	-0.43	0.0135
HDL1-PL	**0.65**	<0.0001	**0.50**	0.0037	0.49	0.0047	0.49	0.0042	0.49	0.0046
HDL2-PL	**0.61**	0.0002	0.42	0.0167	0.40	0.0222	0.43	0.0132	0.41	0.0184
HDL1-apoA-I	**0.62**	0.0001	0.48	0.0051	0.46	0.0075	0.47	0.0061	0.48	0.0059
HDL1-apoA-II	**0.50**	0.0026	0.33	0.0669	0.29	0.1126	0.30	0.0996	0.32	0.0788

Females with MS
(N=31)

	**Unadjusted**		**Model 1**		**Model 2**		**Model 3**		**Model 4**	
**Variable (mg/dL)**	**r**	**p**	**r**	**p**	**r**	**p**	**r**	**p**	**r**	**p**
**LDL**										
LDL3-FC	**0.51**	0.0036	0.47	0.0099	0.47	0.0097	**0.54**	0.0024	**0.51**	0.0049
**HDL**										
HDL2-C	**0.58**	0.0006	**0.57**	0.0012	**0.56**	0.0015	**0.60**	0.0006	**0.60**	0.0007
HDL3-C	**0.56**	0.0011	**0.53**	0.0032	**0.53**	0.0034	**0.59**	0.0007	**0.58**	0.0010
HDL1-FC	**0.51**	0.0036	0.47	0.0094	0.47	0.0105	**0.54**	0.0025	**0.52**	0.0042
HDL2-FC	**0.58**	0.0006	**0.57**	0.0014	**0.57**	0.0013	**0.63**	0.0003	**0.60**	0.0006
HDL3-FC	**0.60**	0.0004	**0.57**	0.0012	**0.57**	0.0013	**0.63**	0.0002	**0.60**	0.0006
HDL2-PL	**0.57**	0.0007	**0.56**	0.0017	**0.55**	0.0021	**0.59**	0.0008	**0.59**	0.0009
HDL3-PL	**0.57**	0.0009	**0.54**	0.0023	**0.54**	0.0024	**0.61**	0.0005	**0.59**	0.0007
HDL2-apoA-I	**0.53**	0.0020	**0.51**	0.0044	**0.50**	0.0053	**0.56**	0.0017	**0.55**	0.0020

Table S4

HDL3-apoA-I	**0.52**	0.0026	0.49	0.0073	0.48	0.0077	**0.56**	0.0015	**0.57**	0.0013

Spearman partial correlation analyses were used to evaluate associations of the serum levels of adiponectin with the serum levels of selected lipoprotein parameters, while accounting for covariates. Spearman correlation coefficients with|r|≥0.5 are depicted in bold. Model 1- adjusted for age and BMI. Model 2 – adjusted for age and waist circumference. Model 3 – adjusted for age and CRP. Model 4 – adjusted for age and IL-6. ApoB, apolipoprotein B; BMI, body mass index; C, cholesterol; FC, free cholesterol; HDL, high-density lipoprotein; IDL, intermediate-density lipoprotein; IL-6, interleukin 6; LDL, low-density lipoprotein; PL, phospholipid; r, Spearman's correlation coefficient; TG, triglyceride, VLDL, very low-density lipoprotein.

Table S4
