# Supplementary material for: Sex-Related Differences in the Associations between Adiponectin and Serum Lipoproteins in Healthy Subjects and Patients with Metabolic Syndrome
Source: Biomedicines. 2024 Sep 1;12(9):1972. doi: 10.3390/biomedicines12091972 (PMC11429094; doi:10.3390/biomedicines12091972)
Supplement: Supplementary file 1 [file biomedicines-12-01972-s001.zip › Table S5.pdf]

Table S5. Correlation analyses between serum levels of adiponectin and clinical and laboratory parameters, performed separately in healthy females and males, as well as females and males with MS.

	Adiponectin (µg/mL)							
	Healthy				MS			
	Female (N=31)		Male (N=34)		Female (N=31)		Male (N=34)	
**Variable**	**r**	**p**	**r**	**p**	**r**	**p**	**r**	**p**
Age (years)	0.16	0.3865	**0.53**	0.0013	-0.03	0.8790	0.20	0.2635
BMI (kg/m²)	-0.23	0.2053	-0.14	0.4286	-0.20	0.2718	-0.11	0.5328
WC (cm)	-0.01	0.9479	-0.31	0.0721	-0.22	0.2280	-0.09	0.5986
HR (beats/min)	-0.14	0.4674	0.08	0.6678	-0.33	0.0744	-0.37	0.0317
SBP (mmHg)	0.43	0.0169	-0.08	0.6477	0.23	0.2175	-0.04	0.8169
DBP (mmHg)	0.47	0.0083	-0.11	0.5217	0.11	0.5598	-0.01	0.9439
MAP (mmHg)	0.44	0.0133	-0.10	0.5922	0.29	0.1134	-0.05	0.7834
Protein (g/L)	-0.16	0.4011	-0.25	0.1486	-0.09	0.6409	-0.26	0.1337
Albumin (g/L)	-0.23	0.2144	-0.35	0.0445	-0.06	0.7387	-0.21	0.2403
Glucose (mmol/L)	0.14	0.4401	0.08	0.6583	-0.31	0.0924	-0.07	0.7114
CRP (µg/mL)	-0.19	0.3094	-0.01	0.9699	0.08	0.6691	-0.35	0.0418
IL-6 (pg/mL)	-0.17	0.3465	0.12	0.4872	0.10	0.5856	-0.04	0.8311
Bilirubin (µmol/L)	-0.08	0.6495	0.14	0.4138	-0.01	0.9553	0.17	0.3459
AST (U/L)	0.12	0.5112	-0.32	0.0608	-0.05	0.7932	-0.17	0.3408
ALT (U/L)	0.16	0.4000	-0.37	0.0307	0.08	0.6752	-0.34	0.0466
AP (U/L)	0.19	0.3072	-0.11	0.5225	-0.14	0.4676	0.11	0.5234
GGT (U/L)	-0.14	0.4431	-0.37	0.0337	-0.23	0.2178	-0.20	0.2459
LDH (U/L)	0.14	0.4375	-0.01	0.9682	0.39	0.0316	0.04	0.8141
Urea (mmol/L)	0.30	0.1046	-0.21	0.2414	0.10	0.6023	-0.14	0.4403
Urate (µmol/L)	-0.21	0.2685	0.15	0.3974	-0.25	0.1792	-0.26	0.1398
Creatinine (µmol/L)	-0.08	0.6620	-0.13	0.4643	0.01	0.9515	-0.22	0.2281
eGFR (mL/min/1.73 m²)	-0.02	0.9356	-0.06	0.7282	-0.02	0.9331	0.12	0.5053

Spearman correlation analyses were used to evaluate associations of the serum levels of adiponectin with clinical and laboratory parameters. Spearman correlation coefficients with $|r| \geq 0.5$ are depicted in bold. ALT, alanine aminotransferase; AP, alkaline phosphatase; AST, aspartate aminotransferase; BMI, body mass index; CK, creatine kinase; CRP, C-reactive protein; DBP, diastolic blood pressure; eGFR, estimated glomerular filtration rate; GGT, gamma-glutamyl transpeptidase; HR, heart rate; IL-6, interleukin 6; LDH, lactate dehydrogenase; MAP, mean arterial pressure; MS, metabolic syndrome patient; SBP, systolic blood pressure; WC, waist circumference.
