# Supplementary material for: Sex-Related Differences in the Associations between Adiponectin and Serum Lipoproteins in Healthy Subjects and Patients with Metabolic Syndrome
Source: Biomedicines. 2024 Sep 1;12(9):1972. doi: 10.3390/biomedicines12091972 (PMC11429094; doi:10.3390/biomedicines12091972)
Supplement: Supplementary file 1 [file biomedicines-12-01972-s001.zip › Table S6.pdf]

Table S6. Correlation analyses of age with serum levels of VLDL, IDL, LDL, and HDL, performed separately in healthy females and males, as well as females and males with MS.

	Age (years)							
	Healthy				MS			
	Female (N=31)		Male (N=34)		Female (N=31)		Male (N=34)	
**Variable (mg/dL)**	**r**	**p**	**r**	**p**	**r**	**p**	**r**	**p**
**VLDL**								
VLDL1-C	0.02	0.9241	-0.49	0.0033	0.01	0.9673	-0.22	0.2018
VLDL2-C	-0.13	0.4786	**-0.53**	0.0013	0.08	0.6654	-0.34	0.0515
VLDL3-C	-0.16	0.3854	**-0.50**	0.0027	0.05	0.8080	-0.40	0.0190
VLDL4-C	-0.12	0.5267	**-0.50**	0.0024	0.01	0.9664	-0.24	0.1781
VLDL5-C	0.19	0.3046	-0.27	0.1201	-0.10	0.5794	0.31	0.0763
VLDL1-FC	-0.03	0.8885	-0.47	0.0046	-0.03	0.8824	-0.24	0.1808
VLDL2-FC	-0.17	0.3566	**-0.52**	0.0015	0.08	0.6784	-0.31	0.0715
VLDL3-FC	-0.16	0.3751	**-0.50**	0.0027	0.10	0.5757	-0.29	0.0975
VLDL4-FC	-0.14	0.4498	-0.47	0.0052	0.01	0.9767	-0.33	0.0590
VLDL5-FC	0.08	0.6638	-0.24	0.1663	-0.20	0.2931	0.05	0.7876
VLDL1-TG	-0.11	0.5528	-0.49	0.0033	0.06	0.7590	-0.19	0.2693
VLDL2-TG	-0.22	0.2243	-0.48	0.0039	0.12	0.5192	-0.27	0.1247
VLDL3-TG	-0.22	0.2309	**-0.50**	0.0029	0.14	0.4502	-0.29	0.1011
VLDL4-TG	-0.21	0.2650	-0.43	0.0115	0.07	0.7004	-0.17	0.3283
VLDL5-TG	0.18	0.3247	-0.22	0.2025	-0.05	0.7825	0.23	0.1939
VLDL1-PL	-0.06	0.7601	**-0.55**	0.0008	0.01	0.9699	-0.21	0.2231
VLDL2-PL	-0.21	0.2537	-0.48	0.0039	0.04	0.8113	-0.29	0.0929
VLDL3-PL	-0.21	0.2636	-0.46	0.0061	0.05	0.7892	-0.31	0.0768
VLDL4-PL	-0.17	0.3495	-0.48	0.0039	0.04	0.8181	-0.22	0.2194
VLDL5-PL	0.11	0.5733	-0.36	0.0383	-0.19	0.3086	0.19	0.2701
VLDL-apoB	-0.17	0.3705	-0.48	0.0043	0.06	0.7336	-0.18	0.2978
**IDL**								

	Age (years)							
	Healthy				MS			
	Female (N=31)		Male (N=34)		Female (N=31)		Male (N=34)	
**Variable (mg/dL)**	**r**	**p**	**r**	**p**	**r**	**p**	**r**	**p**
IDL-C	0.00	0.9810	-0.37	0.0321	-0.11	0.5716	-0.35	0.0397
IDL-FC	-0.04	0.8345	-0.38	0.0283	-0.03	0.8611	-0.36	0.0352
IDL-TG	-0.08	0.6726	-0.42	0.0128	0.03	0.8811	-0.19	0.2845
IDL-PL	-0.07	0.6961	-0.42	0.0125	0.07	0.6924	-0.33	0.0555
IDL-apoB	0.00	0.9931	-0.37	0.0297	-0.13	0.4968	-0.35	0.0410
**LDL**								
LDL1-C	0.29	0.1178	-0.03	0.8692	-0.21	0.2661	-0.03	0.8647
LDL2-C	0.07	0.7038	0.19	0.2773	-0.20	0.2724	-0.06	0.7332
LDL3-C	-0.01	0.9439	0.16	0.3560	-0.21	0.2662	-0.13	0.4575
LDL4-C	-0.10	0.6012	-0.11	0.5178	-0.23	0.2053	-0.20	0.2672
LDL5-C	-0.16	0.3878	-0.32	0.0613	-0.26	0.1548	-0.26	0.1433
LDL6-C	0.14	0.4468	-0.26	0.1430	0.06	0.7495	-0.31	0.0727
LDL1-FC	0.29	0.1149	-0.01	0.9402	-0.16	0.4024	-0.05	0.7943
LDL2-FC	0.07	0.7062	0.24	0.1703	-0.15	0.4336	-0.14	0.4143
LDL3-FC	0.05	0.7767	0.24	0.1809	-0.12	0.5356	-0.17	0.3362
LDL4-FC	-0.07	0.6949	-0.02	0.9077	-0.23	0.2053	-0.17	0.3382
LDL5-FC	-0.12	0.5325	-0.26	0.1419	-0.19	0.3048	-0.26	0.1329
LDL6-FC	0.09	0.6167	-0.14	0.4261	0.03	0.8807	-0.30	0.0802
LDL1-TG	0.11	0.5476	-0.23	0.1849	0.00	0.9940	-0.01	0.9575
LDL2-TG	0.10	0.5920	0.01	0.9512	-0.23	0.2080	-0.16	0.3759
LDL3-TG	0.22	0.2269	0.00	0.9780	-0.24	0.2005	0.05	0.7820
LDL4-TG	-0.09	0.6248	-0.34	0.0504	-0.31	0.0892	-0.24	0.1719
LDL5-TG	-0.10	0.6019	-0.40	0.0178	-0.24	0.1912	-0.31	0.0715
LDL6-TG	0.02	0.9091	-0.11	0.5530	0.05	0.7863	-0.23	0.1881
LDL1-PL	0.30	0.0991	-0.03	0.8713	-0.20	0.2724	0.00	0.9969
LDL2-PL	0.09	0.6490	0.21	0.2318	-0.21	0.2662	-0.05	0.7731
LDL3-PL	-0.02	0.8941	0.18	0.2990	-0.23	0.2110	-0.12	0.5085

	Age (years)							
	Healthy				MS			
	Female (N=31)		Male (N=34)		Female (N=31)		Male (N=34)	
**Variable (mg/dL)**	**r**	**p**	**r**	**p**	**r**	**p**	**r**	**p**
LDL4-PL	-0.07	0.7078	-0.13	0.4778	-0.26	0.1595	-0.19	0.2828
LDL5-PL	-0.14	0.4565	-0.31	0.0728	-0.29	0.1074	-0.25	0.1463
LDL6-PL	0.15	0.4106	-0.19	0.2860	0.04	0.8400	-0.32	0.0617
LDL1-apoB	0.30	0.1007	-0.01	0.9660	-0.19	0.2934	0.00	0.9931
LDL2-apoB	0.07	0.7110	0.20	0.2526	-0.24	0.2013	-0.09	0.6312
LDL3-apoB	-0.05	0.7925	0.15	0.3989	-0.24	0.1881	-0.15	0.3820
LDL4-apoB	-0.11	0.5608	-0.19	0.2917	-0.29	0.1171	-0.22	0.2087
LDL5-apoB	-0.14	0.4558	-0.35	0.0419	-0.30	0.1013	-0.29	0.0988
LDL6-apoB	0.10	0.6027	-0.26	0.1394	0.06	0.7409	-0.30	0.0876
**HDL**								
HDL1-C	0.10	0.5771	**0.52**	0.0016	-0.02	0.9346	0.08	0.6623
HDL2-C	0.15	0.4056	**0.64**	<0.0001	-0.21	0.2553	0.00	0.9952
HDL3-C	0.30	0.0957	**0.61**	0.0001	-0.11	0.5554	0.00	0.9904
HDL4-C	0.06	0.7453	0.19	0.2825	0.28	0.1221	0.02	0.8980
HDL1-FC	0.07	0.6917	**0.51**	0.0018	-0.06	0.7344	-0.24	0.1748
HDL2-FC	0.14	0.4578	**0.57**	0.0004	-0.21	0.2456	-0.10	0.5804
HDL3-FC	0.08	0.6864	**0.56**	0.0006	-0.07	0.6891	-0.09	0.6208
HDL4-FC	0.02	0.9052	0.26	0.1448	0.19	0.3047	-0.11	0.5387
HDL1-TG	0.09	0.6337	0.15	0.3872	0.02	0.8973	-0.10	0.5876
HDL2-TG	0.07	0.6957	-0.07	0.6777	-0.13	0.5005	-0.17	0.3230
HDL3-TG	0.01	0.9400	-0.21	0.2265	-0.24	0.1877	-0.14	0.4239
HDL4-TG	-0.08	0.6849	-0.45	0.0080	-0.05	0.8030	-0.18	0.3172
HDL1-PL	0.12	0.5189	**0.58**	0.0003	-0.11	0.5435	0.12	0.5080
HDL2-PL	0.21	0.2491	**0.64**	0.0001	-0.20	0.2827	0.05	0.7969
HDL3-PL	0.28	0.1246	**0.61**	0.0001	-0.19	0.2980	0.05	0.7665
HDL4-PL	0.12	0.5253	0.34	0.0480	0.09	0.6153	-0.02	0.9000

Table S6

	Age (years)							
	Healthy				MS			
	Female (N=31)		Male (N=34)		Female (N=31)		Male (N=34)	
**Variable (mg/dL)**	**r**	**p**	**r**	**p**	**r**	**p**	**r**	**p**
HDL1-apoA-I	0.04	0.8185	**0.53**	0.0013	-0.05	0.7996	-0.01	0.9657
HDL2-apoA-I	0.17	0.3669	**0.64**	<0.0001	-0.21	0.2621	-0.04	0.8230
HDL3-apoA-I	0.28	0.1312	**0.60**	0.0002	-0.11	0.5716	-0.04	0.8083
HDL4-apoA-I	0.02	0.9250	0.12	0.5162	0.27	0.1383	-0.09	0.6093
HDL1-apoA-II	0.01	0.9431	**0.55**	0.0008	-0.20	0.2842	-0.09	0.5983
HDL2-apoA-II	0.07	0.6901	0.43	0.0110	-0.29	0.1177	-0.21	0.2247
HDL3-apoA-II	0.08	0.6734	0.28	0.1071	-0.24	0.1900	-0.20	0.2479
HDL4-apoA-II	-0.07	0.7122	0.00	0.9938	0.27	0.1489	-0.21	0.2358

Spearman correlation analyses were used to evaluate associations of age with the serum levels of VLDL, IDL, LDL, and HDL. Spearman correlation coefficients with $|r| \geq 0.5$ are depicted in bold. ApoA-I, apolipoprotein A-I, apoA-II, apolipoprotein A-II; apoB, apolipoprotein B; C, cholesterol; FC, free cholesterol; HDL, high-density lipoprotein; IDL, intermediate-density lipoprotein; LDL, low-density lipoprotein; MS; metabolic syndrome patient; VLDL, very low-density lipoprotein; PL, phospholipid; TG, triglyceride.
