# Supplementary material for: Sex-Related Differences in the Associations between Adiponectin and Serum Lipoproteins in Healthy Subjects and Patients with Metabolic Syndrome
Source: Biomedicines. 2024 Sep 1;12(9):1972. doi: 10.3390/biomedicines12091972 (PMC11429094; doi:10.3390/biomedicines12091972)
Supplement: Supplementary file 1 [file biomedicines-12-01972-s001.zip › Table S7.pdf]

Table S7. Correlation analyses of BMI with serum levels of VLDL, IDL, LDL, and HDL, performed separately in healthy females and males, as well as females and males with MS.

	BMI (kg/m²)							
	Healthy				MS			
	Female (N=31)		Male (N=34)		Female (N=31)		Male (N=34)	
**Variable (mg/dL)**	**r**	**p**	**r**	**p**	**r**	**p**	**r**	**p**
**VLDL**								
VLDL1-C	0.33	0.0676	0.31	0.0759	-0.01	0.9622	-0.27	0.1212
VLDL2-C	0.23	0.2086	0.35	0.0425	0.05	0.7993	-0.28	0.1074
VLDL3-C	0.26	0.1527	0.33	0.0604	0.09	0.6386	-0.26	0.1394
VLDL4-C	0.16	0.3884	0.34	0.0516	0.01	0.9611	-0.16	0.3564
VLDL5-C	0.29	0.1189	0.25	0.1522	-0.18	0.3396	0.02	0.8929
VLDL1-FC	0.33	0.0662	0.32	0.0645	-0.01	0.9749	-0.27	0.1227
VLDL2-FC	0.28	0.1301	0.37	0.0292	0.05	0.7703	-0.29	0.0962
VLDL3-FC	0.29	0.1092	0.30	0.0823	0.09	0.6417	-0.33	0.0605
VLDL4-FC	0.29	0.1179	0.31	0.0723	-0.05	0.7728	-0.18	0.3147
VLDL5-FC	0.10	0.5931	0.05	0.7772	-0.11	0.5495	0.19	0.2923
VLDL1-TG	0.27	0.1414	0.26	0.1443	0.03	0.8581	-0.27	0.1278
VLDL2-TG	0.21	0.2679	0.31	0.0730	0.08	0.6747	-0.37	0.0331
VLDL3-TG	0.20	0.2728	0.32	0.0675	0.05	0.7940	-0.35	0.0434
VLDL4-TG	0.23	0.2066	0.22	0.2108	0.04	0.8411	-0.20	0.2594
VLDL5-TG	0.15	0.4230	0.11	0.5459	-0.09	0.6320	0.27	0.1224
VLDL1-PL	0.30	0.1067	0.36	0.0360	0.06	0.7279	-0.28	0.1144
VLDL2-PL	0.20	0.2795	0.34	0.0482	0.12	0.5091	-0.36	0.0390
VLDL3-PL	0.25	0.1778	0.27	0.1194	0.09	0.6128	-0.33	0.0575
VLDL4-PL	0.27	0.1409	0.33	0.0587	0.02	0.9356	-0.16	0.3647
VLDL5-PL	0.34	0.0590	0.26	0.1394	-0.12	0.5125	0.09	0.6175
VLDL-apoB	0.28	0.1233	0.26	0.1334	0.00	0.9957	-0.28	0.1098
**IDL**								

	BMI (kg/m²)							
	Healthy				MS			
	Female (N=31)		Male (N=34)		Female (N=31)		Male (N=34)	
**Variable (mg/dL)**	**r**	**p**	**r**	**p**	**r**	**p**	**r**	**p**
IDL-C	-0.01	0.9387	0.33	0.0594	-0.02	0.9279	-0.15	0.4009
IDL-FC	0.02	0.9180	0.33	0.0573	-0.05	0.8057	-0.16	0.3693
IDL-TG	0.26	0.1527	0.25	0.1607	-0.10	0.6090	-0.26	0.1447
IDL-PL	0.01	0.9614	0.26	0.1308	-0.16	0.3785	-0.22	0.2120
IDL-apoB	-0.11	0.5367	0.22	0.2151	-0.07	0.7165	-0.08	0.6472
**LDL**								
LDL1-C	-0.26	0.1520	0.31	0.0738	-0.35	0.0561	0.12	0.4945
LDL2-C	-0.37	0.0423	0.14	0.4380	-0.12	0.5252	0.40	0.0189
LDL3-C	-0.28	0.1261	0.20	0.2605	-0.31	0.0930	0.10	0.5673
LDL4-C	0.03	0.8837	0.31	0.0787	-0.38	0.0382	-0.07	0.6896
LDL5-C	-0.11	0.5482	0.14	0.4172	-0.12	0.5083	-0.19	0.2920
LDL6-C	-0.31	0.0938	-0.12	0.5031	0.14	0.4640	-0.02	0.9126
LDL1-FC	-0.24	0.1952	0.27	0.1216	-0.39	0.0315	0.16	0.3546
LDL2-FC	-0.38	0.0384	0.01	0.9610	-0.17	0.3699	0.46	0.0059
LDL3-FC	-0.29	0.1198	0.03	0.8523	-0.34	0.0597	0.21	0.2219
LDL4-FC	-0.01	0.9646	0.21	0.2361	-0.38	0.0342	-0.02	0.9228
LDL5-FC	-0.13	0.4834	0.10	0.5651	-0.20	0.2904	-0.18	0.2968
LDL6-FC	-0.34	0.0635	-0.19	0.2943	0.11	0.5694	-0.04	0.8168
LDL1-TG	-0.10	0.5865	0.17	0.3252	-0.15	0.4127	0.20	0.2566
LDL2-TG	-0.27	0.1449	0.20	0.2595	-0.16	0.3908	0.35	0.0394
LDL3-TG	-0.39	0.0305	0.08	0.6613	-0.19	0.2976	0.30	0.0875
LDL4-TG	0.10	0.5753	0.23	0.1833	-0.13	0.4727	-0.12	0.4905
LDL5-TG	0.04	0.8158	0.17	0.3372	-0.07	0.7043	-0.12	0.4912
LDL6-TG	-0.28	0.1233	-0.23	0.1883	0.33	0.0655	-0.10	0.5745
LDL1-PL	-0.27	0.1422	0.29	0.1008	-0.33	0.0698	0.18	0.3121
LDL2-PL	-0.34	0.0649	0.13	0.4599	-0.16	0.3917	0.41	0.0170
LDL3-PL	-0.26	0.1540	0.21	0.2397	-0.29	0.1091	0.13	0.4695

	BMI (kg/m²)							
	Healthy				MS			
	Female (N=31)		Male (N=34)		Female (N=31)		Male (N=34)	
**Variable (mg/dL)**	**r**	**p**	**r**	**p**	**r**	**p**	**r**	**p**
LDL4-PL	0.05	0.8091	0.31	0.0775	-0.38	0.0367	-0.08	0.6581
LDL5-PL	-0.09	0.6170	0.15	0.3897	-0.10	0.5732	-0.17	0.3323
LDL6-PL	-0.33	0.0734	-0.18	0.2980	0.11	0.5599	-0.02	0.9052
LDL1-apoB	-0.31	0.0925	0.28	0.1143	-0.33	0.0741	0.22	0.2172
LDL2-apoB	-0.37	0.0380	0.10	0.5881	-0.13	0.4712	0.43	0.0128
LDL3-apoB	-0.26	0.1619	0.23	0.1950	-0.28	0.1301	0.11	0.5277
LDL4-apoB	0.02	0.9232	0.31	0.0747	-0.36	0.0445	-0.11	0.5327
LDL5-apoB	-0.09	0.6200	0.16	0.3722	-0.07	0.7246	-0.16	0.3647
LDL6-apoB	-0.24	0.1861	-0.10	0.5798	0.14	0.4447	-0.04	0.8346
**HDL**								
HDL1-C	-0.26	0.1579	-0.26	0.1450	-0.20	0.2844	0.47	0.0060
HDL2-C	-0.22	0.2236	-0.34	0.0513	-0.16	0.3833	0.42	0.0137
HDL3-C	-0.09	0.6386	-0.39	0.0208	-0.33	0.0683	0.32	0.0670
HDL4-C	-0.19	0.3017	-0.15	0.4053	-0.18	0.3402	0.01	0.9713
HDL1-FC	-0.26	0.1606	-0.29	0.0962	-0.28	0.1211	0.27	0.1181
HDL2-FC	-0.18	0.3294	-0.30	0.0805	-0.29	0.1137	0.20	0.2500
HDL3-FC	-0.30	0.1061	-0.38	0.0277	-0.33	0.0672	0.02	0.9303
HDL4-FC	-0.14	0.4365	-0.15	0.3853	-0.27	0.1394	-0.07	0.7139
HDL1-TG	0.00	0.9931	-0.33	0.0586	-0.19	0.2978	0.13	0.4557
HDL2-TG	0.27	0.1449	-0.13	0.4534	-0.17	0.3618	0.07	0.6884
HDL3-TG	0.35	0.0536	0.02	0.9047	-0.17	0.3682	-0.02	0.9148
HDL4-TG	0.23	0.2038	0.11	0.5482	-0.22	0.2295	-0.25	0.1584
HDL1-PL	-0.21	0.2500	-0.29	0.0967	-0.22	0.2287	0.47	0.0055
HDL2-PL	-0.12	0.5266	-0.34	0.0510	-0.19	0.2932	0.42	0.0143
HDL3-PL	-0.07	0.7012	-0.41	0.0163	-0.31	0.0880	0.25	0.1596
HDL4-PL	-0.08	0.6763	-0.27	0.1267	-0.28	0.1334	0.05	0.7968

	BMI (kg/m²)							
	Healthy				MS			
	Female (N=31)		Male (N=34)		Female (N=31)		Male (N=34)	
**Variable (mg/dL)**	**r**	**p**	**r**	**p**	**r**	**p**	**r**	**p**
HDL1-apoA-I	-0.24	0.1906	-0.29	0.0987	-0.23	0.2227	0.39	0.0215
HDL2-apoA-I	-0.25	0.1674	-0.49	0.0033	-0.20	0.2897	0.45	0.0081
HDL3-apoA-I	-0.04	0.8421	-0.40	0.0196	-0.39	0.0319	0.37	0.0299
HDL4-apoA-I	-0.09	0.6170	-0.11	0.5437	-0.30	0.1003	-0.05	0.7758
HDL1-apoA-II	-0.27	0.1468	-0.38	0.0277	-0.18	0.3226	0.22	0.2101
HDL2-apoA-II	-0.21	0.2542	-0.33	0.0592	-0.16	0.3800	0.26	0.1370
HDL3-apoA-II	0.06	0.7574	-0.20	0.2649	-0.30	0.0966	0.16	0.3712
HDL4-apoA-II	-0.15	0.4307	-0.02	0.8983	-0.27	0.1389	-0.14	0.4329

Spearman correlation analyses were used to evaluate associations of BMI with the serum levels of VLDL, IDL, LDL, and HDL. Spearman correlation coefficients with|r|≥0.5 are depicted in bold. ApoA-I, apolipoprotein A-I, apoA-II, apolipoprotein A-II; apoB, apolipoprotein B; BMI, body mass index; C, cholesterol; FC, free cholesterol; HDL, high-density lipoprotein; IDL, intermediate-density lipoprotein; LDL, low-density lipoprotein; MS; metabolic syndrome patient; VLDL, very low-density lipoprotein; PL, phospholipid; TG, triglyceride.

Table S7
