# Supplementary material for: Sex-Related Differences in the Associations between Adiponectin and Serum Lipoproteins in Healthy Subjects and Patients with Metabolic Syndrome
Source: Biomedicines. 2024 Sep 1;12(9):1972. doi: 10.3390/biomedicines12091972 (PMC11429094; doi:10.3390/biomedicines12091972)
Supplement: Supplementary file 1 [file biomedicines-12-01972-s001.zip › Table S8.pdf]

Table S8. Correlation analyses of waist circumference with serum levels of VLDL, IDL, LDL, and HDL, performed separately in healthy females and males, as well as females and males with MS.

	Waist circumference (cm)							
	Healthy				MS			
	Female (N=31)		Male (N=34)		Female (N=31)		Male (N=34)	
**Variable (mg/dL)**	**r**	**p**	**r**	**p**	**r**	**p**	**r**	**p**
**VLDL**								
VLDL1-C	0.10	0.5792	0.33	0.0536	-0.04	0.8230	-0.38	0.0269
VLDL2-C	-0.01	0.9552	0.44	0.0093	0.03	0.8551	-0.36	0.0391
VLDL3-C	-0.03	0.8806	0.42	0.0132	0.08	0.6527	-0.29	0.0947
VLDL4-C	-0.09	0.6400	0.48	0.0041	0.00	0.9957	-0.22	0.2148
VLDL5-C	0.26	0.1640	0.18	0.3114	-0.16	0.3913	0.05	0.7578
VLDL1-FC	0.09	0.6404	0.40	0.0187	-0.02	0.9125	-0.37	0.0334
VLDL2-FC	0.02	0.9092	0.44	0.0088	0.07	0.6925	-0.36	0.0389
VLDL3-FC	0.02	0.8947	0.40	0.0198	0.08	0.6613	-0.37	0.0316
VLDL4-FC	0.00	0.9948	0.42	0.0129	-0.08	0.6656	-0.22	0.2131
VLDL5-FC	-0.05	0.7827	0.08	0.6425	-0.23	0.2055	0.11	0.5376
VLDL1-TG	0.05	0.8053	0.30	0.0846	0.01	0.9485	-0.33	0.0531
VLDL2-TG	-0.01	0.9728	0.40	0.0186	0.09	0.6356	-0.43	0.0119
VLDL3-TG	-0.02	0.9200	0.42	0.0141	0.10	0.5997	-0.38	0.0268
VLDL4-TG	-0.02	0.8943	0.38	0.0285	0.07	0.7187	-0.21	0.2376
VLDL5-TG	0.17	0.3667	0.08	0.6561	-0.13	0.4724	0.19	0.2745
VLDL1-PL	0.05	0.7761	0.39	0.0223	0.05	0.7976	-0.34	0.0515
VLDL2-PL	-0.05	0.8095	0.45	0.0083	0.12	0.5165	-0.41	0.0173
VLDL3-PL	-0.01	0.9462	0.40	0.0183	0.11	0.5494	-0.36	0.0340
VLDL4-PL	0.00	0.9797	0.46	0.0064	0.03	0.8801	-0.19	0.2925
VLDL5-PL	0.23	0.2095	0.22	0.2128	-0.14	0.4531	0.10	0.5630
VLDL-apoB	0.01	0.9423	0.41	0.0163	0.01	0.9399	-0.35	0.0403
**IDL**								

	Waist circumference (cm)							
	Healthy				MS			
	Female (N=31)		Male (N=34)		Female (N=31)		Male (N=34)	
Variable (mg/dL)	r	p	r	p	r	p	r	p
IDL-C	-0.24	0.1843	0.45	0.0069	-0.06	0.7349	-0.22	0.2013
IDL-FC	-0.23	0.2127	0.47	0.0051	-0.09	0.6457	-0.23	0.1887
IDL-TG	0.05	0.8012	0.32	0.0615	-0.09	0.6122	-0.34	0.0457
IDL-PL	-0.19	0.3022	0.38	0.0248	-0.16	0.3942	-0.31	0.0789
IDL-apoB	-0.33	0.0696	0.40	0.0182	-0.14	0.4658	-0.15	0.4100
**LDL**								
LDL1-C	-0.18	0.3279	0.22	0.2143	-0.35	0.0516	0.05	0.7994
LDL2-C	-0.17	0.3583	0.00	0.9856	-0.11	0.5465	0.40	0.0208
LDL3-C	-0.24	0.1843	0.04	0.8085	-0.30	0.0970	0.15	0.3938
LDL4-C	-0.17	0.3647	0.29	0.0980	-0.30	0.0994	-0.01	0.9576
LDL5-C	-0.27	0.1424	0.28	0.1095	-0.09	0.6333	-0.17	0.3338
LDL6-C	-0.33	0.0742	0.06	0.7566	0.16	0.3914	-0.14	0.4415
LDL1-FC	-0.14	0.4604	0.18	0.3014	-0.38	0.0352	0.08	0.6731
LDL2-FC	-0.17	0.3497	-0.12	0.4908	-0.13	0.4901	0.41	0.0149
LDL3-FC	-0.16	0.3848	-0.09	0.6091	-0.30	0.1042	0.24	0.1738
LDL4-FC	-0.13	0.4732	0.15	0.3872	-0.31	0.0867	0.02	0.8996
LDL5-FC	-0.23	0.2098	0.24	0.1691	-0.17	0.3511	-0.15	0.3880
LDL6-FC	-0.24	0.1965	0.01	0.9750	0.14	0.4420	-0.14	0.4184
LDL1-TG	-0.27	0.1349	0.38	0.0288	-0.19	0.3191	0.04	0.8240
LDL2-TG	-0.35	0.0515	0.26	0.1366	-0.22	0.2391	0.25	0.1526
LDL3-TG	-0.31	0.0870	0.12	0.4840	-0.32	0.0791	0.26	0.1310
LDL4-TG	-0.13	0.4728	0.39	0.0234	-0.17	0.3591	-0.07	0.6974
LDL5-TG	-0.15	0.4063	0.37	0.0311	-0.06	0.7643	-0.17	0.3471
LDL6-TG	-0.27	0.1469	-0.02	0.9210	0.30	0.0993	-0.16	0.3595
LDL1-PL	-0.18	0.3320	0.21	0.2261	-0.36	0.0436	0.10	0.5695
LDL2-PL	-0.14	0.4407	-0.01	0.9493	-0.15	0.4190	0.41	0.0167
LDL3-PL	-0.26	0.1646	0.05	0.7787	-0.30	0.1045	0.18	0.3203

	Waist circumference (cm)							
	Healthy				MS			
	Female (N=31)		Male (N=34)		Female (N=31)		Male (N=34)	
Variable (mg/dL)	r	p	r	p	r	p	r	p
LDL4-PL	-0.15	0.4176	0.30	0.0798	-0.33	0.0689	-0.02	0.9295
LDL5-PL	-0.25	0.1744	0.28	0.1063	-0.07	0.7010	-0.15	0.3827
LDL6-PL	-0.28	0.1203	0.00	0.9918	0.11	0.5444	-0.12	0.4858
LDL1-apoB	-0.21	0.2516	0.23	0.1895	-0.36	0.0470	0.12	0.4897
LDL2-apoB	-0.19	0.2989	-0.03	0.8507	-0.16	0.3936	0.39	0.0209
LDL3-apoB	-0.29	0.1083	0.09	0.5991	-0.30	0.1025	0.15	0.4022
LDL4-apoB	-0.20	0.2824	0.34	0.0495	-0.33	0.0714	-0.05	0.7617
LDL5-apoB	-0.26	0.1625	0.30	0.0890	-0.07	0.7090	-0.17	0.3285
LDL6-apoB	-0.31	0.0919	0.08	0.6671	0.15	0.4184	-0.16	0.3770
**HDL**								
HDL1-C	-0.02	0.8960	-0.33	0.0541	-0.20	0.2883	0.38	0.0286
HDL2-C	0.05	0.7728	-0.43	0.0115	-0.30	0.1065	0.43	0.0103
HDL3-C	0.11	0.5403	-0.47	0.0048	-0.42	0.0176	0.36	0.0381
HDL4-C	-0.11	0.5577	-0.22	0.2133	-0.08	0.6874	0.13	0.4769
HDL1-FC	-0.04	0.8475	-0.42	0.0132	-0.36	0.0491	0.27	0.1285
HDL2-FC	0.02	0.9307	-0.39	0.0239	-0.48	0.0061	0.27	0.1266
HDL3-FC	-0.14	0.4659	-0.44	0.0096	-0.37	0.0384	0.10	0.5648
HDL4-FC	-0.11	0.5687	-0.19	0.2904	-0.12	0.5321	0.05	0.7930
HDL1-TG	-0.01	0.9660	-0.29	0.0960	-0.15	0.4240	-0.02	0.9027
HDL2-TG	0.14	0.4556	-0.06	0.7539	-0.17	0.3500	-0.06	0.7169
HDL3-TG	0.10	0.5909	0.17	0.3237	-0.17	0.3585	-0.11	0.5406
HDL4-TG	-0.05	0.8049	0.26	0.1386	-0.10	0.6088	-0.29	0.1005
HDL1-PL	-0.02	0.9303	-0.35	0.0404	-0.31	0.0911	0.42	0.0139
HDL2-PL	0.12	0.5266	-0.39	0.0211	-0.36	0.0478	0.44	0.0100
HDL3-PL	0.11	0.5617	-0.45	0.0070	-0.46	0.0098	0.31	0.0757
HDL4-PL	-0.02	0.8985	-0.30	0.0843	-0.22	0.2308	0.17	0.3506

Table S8

	Waist circumference (cm)							
	Healthy				MS			
	Female (N=31)		Male (N=34)		Female (N=31)		Male (N=34)	
Variable (mg/dL)	r	p	r	p	r	p	r	p
HDL1-apoA-I	-0.07	0.7002	-0.37	0.0311	-0.25	0.1711	0.33	0.0544
HDL2-apoA-I	-0.06	0.7506	**-0.54**	0.0010	-0.33	0.0742	0.42	0.0126
HDL3-apoA-I	0.08	0.6510	-0.49	0.0031	-0.48	0.0065	0.36	0.0364
HDL4-apoA-I	-0.10	0.5972	-0.15	0.3991	-0.14	0.4658	0.04	0.8254
HDL1-apoA-II	-0.17	0.3507	-0.37	0.0293	-0.33	0.0738	0.19	0.2913
HDL2-apoA-II	-0.14	0.4471	-0.30	0.0896	-0.35	0.0514	0.22	0.2105
HDL3-apoA-II	-0.04	0.8492	-0.10	0.5738	-0.46	0.0085	0.10	0.5613
HDL4-apoA-II	-0.19	0.3059	-0.02	0.8995	-0.14	0.4651	-0.07	0.6775

Spearman correlation analyses were used to evaluate associations of BMI with the serum levels of VLDL, IDL, LDL, and HDL. Spearman correlation coefficients with|r|≥0.5 are depicted in bold. ApoA-I, apolipoprotein A-I, apoA-II, apolipoprotein A-II; apoB, apolipoprotein B; C, cholesterol; FC, free cholesterol; HDL, high-density lipoprotein; IDL, intermediate-density lipoprotein; LDL, low-density lipoprotein; MS; metabolic syndrome patient; VLDL, very low-density lipoprotein; PL, phospholipid; TG, triglyceride.
