# Supplementary material for: Sex-Related Differences in the Associations between Adiponectin and Serum Lipoproteins in Healthy Subjects and Patients with Metabolic Syndrome
Source: Biomedicines. 2024 Sep 1;12(9):1972. doi: 10.3390/biomedicines12091972 (PMC11429094; doi:10.3390/biomedicines12091972)
Supplement: Supplementary file 1 [file biomedicines-12-01972-s001.zip › Table S9.pdf]

Table S9. Correlation analyses of IL-6 with serum levels of VLDL, IDL, LDL, and HDL, performed separately in healthy females and males, as well as females and males with MS.

	IL-6 (pg/mL)							
	Healthy				MS			
	Female (N=31)		Male (N=34)		Female (N=31)		Male (N=34)	
**Variable (mg/dL)**	**r**	**p**	**r**	**p**	**r**	**p**	**r**	**p**
**VLDL**								
VLDL1-C	0.17	0.3540	0.17	0.3256	-0.35	0.0548	-0.43	0.0104
VLDL2-C	0.27	0.1398	0.20	0.2647	-0.24	0.2030	-0.40	0.0190
VLDL3-C	0.31	0.0863	0.18	0.3165	-0.24	0.1881	-0.38	0.0279
VLDL4-C	0.31	0.0906	0.16	0.3551	-0.26	0.1599	-0.30	0.0869
VLDL5-C	0.07	0.6945	0.07	0.7119	-0.30	0.0995	0.16	0.3633
VLDL1-FC	0.20	0.2847	0.20	0.2655	-0.37	0.0403	-0.46	0.0063
VLDL2-FC	0.19	0.2984	0.18	0.3090	-0.29	0.1103	-0.40	0.0199
VLDL3-FC	0.19	0.3094	0.14	0.4216	-0.27	0.1381	-0.45	0.0073
VLDL4-FC	0.27	0.1425	0.15	0.3867	-0.34	0.0605	-0.33	0.0602
VLDL5-FC	-0.15	0.4135	-0.18	0.3059	-0.30	0.1046	-0.12	0.4867
VLDL1-TG	0.15	0.4102	0.10	0.5617	-0.34	0.0614	-0.46	0.0061
VLDL2-TG	0.19	0.2936	0.17	0.3374	-0.25	0.1763	-0.43	0.0119
VLDL3-TG	0.20	0.2739	0.13	0.4598	-0.25	0.1681	-0.41	0.0162
VLDL4-TG	0.14	0.4479	0.09	0.6087	-0.27	0.1354	-0.33	0.0554
VLDL5-TG	-0.04	0.8399	-0.13	0.4548	-0.29	0.1166	0.05	0.7670
VLDL1-PL	0.21	0.2648	0.13	0.4567	-0.29	0.1146	-0.43	0.0102
VLDL2-PL	0.23	0.2124	0.21	0.2299	-0.22	0.2249	-0.44	0.0084
VLDL3-PL	0.18	0.3429	0.15	0.4106	-0.26	0.1533	-0.42	0.0132
VLDL4-PL	0.20	0.2870	0.17	0.3312	-0.29	0.1160	-0.29	0.1011
VLDL5-PL	0.05	0.7979	0.07	0.7013	-0.31	0.0892	0.15	0.3851
VLDL-apoB	0.14	0.4427	0.11	0.5266	-0.38	0.0374	-0.38	0.0254
**IDL**								

	IL-6 (pg/mL)							
	Healthy				MS			
	Female (N=31)		Male (N=34)		Female (N=31)		Male (N=34)	
**Variable (mg/dL)**	**r**	**p**	**r**	**p**	**r**	**p**	**r**	**p**
IDL-C	0.39	0.0297	0.18	0.3086	-0.25	0.1793	-0.28	0.1149
IDL-FC	0.39	0.0297	0.20	0.2617	-0.29	0.1170	-0.30	0.0830
IDL-TG	0.16	0.3851	0.10	0.5623	-0.43	0.0166	-0.43	0.0120
IDL-PL	0.39	0.0282	0.17	0.3365	-0.38	0.0371	-0.34	0.0471
IDL-apoB	0.32	0.0773	0.06	0.7194	-0.32	0.0839	-0.22	0.2163
**LDL**								
LDL1-C	0.29	0.1105	0.04	0.8112	-0.10	0.5826	0.16	0.3748
LDL2-C	-0.01	0.9588	-0.08	0.6504	0.20	0.2722	0.48	0.0041
LDL3-C	0.23	0.2178	-0.02	0.9227	0.01	0.9408	0.36	0.0345
LDL4-C	0.35	0.0564	0.04	0.8373	-0.06	0.7670	0.18	0.3072
LDL5-C	0.25	0.1757	-0.05	0.7689	-0.03	0.8547	0.06	0.7261
LDL6-C	0.28	0.1325	-0.04	0.8026	0.10	0.6044	-0.20	0.2543
LDL1-FC	0.33	0.0729	0.04	0.8193	-0.14	0.4527	0.16	0.3577
LDL2-FC	0.05	0.8076	-0.13	0.4809	0.16	0.3852	0.43	0.0105
LDL3-FC	0.26	0.1511	-0.08	0.6491	0.02	0.9254	0.41	0.0171
LDL4-FC	0.36	0.0493	-0.07	0.7148	-0.04	0.8210	0.22	0.2022
LDL5-FC	0.30	0.0956	-0.09	0.6111	0.03	0.8742	0.07	0.6807
LDL6-FC	0.32	0.0829	-0.08	0.6404	0.15	0.4066	-0.17	0.3269
LDL1-TG	0.23	0.2073	0.09	0.6001	-0.29	0.1138	-0.03	0.8833
LDL2-TG	0.15	0.4135	0.06	0.7551	-0.07	0.7091	0.29	0.0948
LDL3-TG	0.06	0.7541	-0.21	0.2417	0.07	0.7188	0.47	0.0054
LDL4-TG	0.28	0.1273	0.10	0.5711	0.06	0.7587	0.20	0.2599
LDL5-TG	0.18	0.3299	0.11	0.5451	-0.11	0.5670	-0.09	0.6012
LDL6-TG	0.00	0.9789	-0.05	0.7840	0.28	0.1293	-0.22	0.2100
LDL1-PL	0.29	0.1161	-0.01	0.9678	-0.09	0.6258	0.21	0.2397
LDL2-PL	0.03	0.8640	-0.08	0.6541	0.17	0.3491	**0.52**	0.0016
LDL3-PL	0.23	0.2224	-0.03	0.8826	0.02	0.9014	0.38	0.0252

	IL-6 (pg/mL)							
	Healthy				MS			
	Female (N=31)		Male (N=34)		Female (N=31)		Male (N=34)	
**Variable (mg/dL)**	**r**	**p**	**r**	**p**	**r**	**p**	**r**	**p**
LDL4-PL	0.40	0.0272	0.04	0.8076	-0.06	0.7326	0.17	0.3242
LDL5-PL	0.29	0.1126	-0.04	0.8172	-0.02	0.9356	0.08	0.6625
LDL6-PL	0.20	0.2894	-0.08	0.6635	0.10	0.5775	-0.21	0.2442
LDL1-apoB	0.25	0.1761	0.03	0.8595	-0.10	0.6052	0.18	0.3188
LDL2-apoB	0.01	0.9536	-0.11	0.5420	0.11	0.5424	**0.52**	0.0017
LDL3-apoB	0.24	0.2016	-0.01	0.9712	0.01	0.9382	0.37	0.0333
LDL4-apoB	0.39	0.0324	0.08	0.6729	-0.11	0.5554	0.14	0.4341
LDL5-apoB	0.28	0.1296	-0.03	0.8785	-0.01	0.9785	0.07	0.7068
LDL6-apoB	0.21	0.2572	-0.02	0.8921	0.03	0.8674	-0.21	0.2371
**HDL**								
HDL1-C	-0.22	0.2439	0.05	0.7774	-0.07	0.7083	0.16	0.3527
HDL2-C	-0.15	0.4343	0.00	0.9873	-0.02	0.9254	0.26	0.1346
HDL3-C	0.15	0.4362	-0.17	0.3486	-0.12	0.5149	0.19	0.2850
HDL4-C	0.21	0.2508	-0.27	0.1288	-0.10	0.5976	0.13	0.4602
HDL1-FC	-0.19	0.3191	0.00	0.9794	-0.06	0.7497	0.16	0.3710
HDL2-FC	-0.01	0.9527	-0.09	0.6186	-0.03	0.8666	0.18	0.2974
HDL3-FC	0.22	0.2426	-0.30	0.0901	0.00	0.9914	0.12	0.5001
HDL4-FC	0.34	0.0644	-0.25	0.1567	-0.02	0.9112	0.08	0.6690
HDL1-TG	-0.17	0.3522	0.06	0.7551	-0.21	0.2675	-0.12	0.5146
HDL2-TG	-0.01	0.9373	0.07	0.7058	-0.21	0.2681	-0.08	0.6550
HDL3-TG	0.18	0.3407	0.07	0.6895	-0.26	0.1512	-0.15	0.3857
HDL4-TG	0.26	0.1634	-0.11	0.5379	-0.42	0.0172	-0.35	0.0457
HDL1-PL	-0.24	0.1995	0.08	0.6553	-0.07	0.6947	0.17	0.3300
HDL2-PL	-0.22	0.2408	0.02	0.9309	-0.03	0.8759	0.21	0.2280
HDL3-PL	0.09	0.6298	-0.19	0.2838	-0.13	0.4753	0.17	0.3252
HDL4-PL	0.34	0.0600	-0.24	0.1788	-0.17	0.3722	0.13	0.4756

	IL-6 (pg/mL)							
	Healthy				MS			
	Female (N=31)		Male (N=34)		Female (N=31)		Male (N=34)	
**Variable (mg/dL)**	**r**	**p**	**r**	**p**	**r**	**p**	**r**	**p**
HDL1-apoA-I	-0.27	0.1457	0.01	0.9644	-0.05	0.8060	0.10	0.5761
HDL2-apoA-I	-0.27	0.1447	-0.19	0.2849	-0.08	0.6618	0.18	0.2956
HDL3-apoA-I	0.16	0.4027	-0.17	0.3313	-0.25	0.1750	0.13	0.4466
HDL4-apoA-I	0.31	0.0929	-0.16	0.3568	-0.24	0.1874	0.01	0.9378
HDL1-apoA-II	-0.33	0.0724	-0.08	0.6484	-0.05	0.7719	-0.12	0.5131
HDL2-apoA-II	-0.24	0.2002	-0.15	0.4100	-0.09	0.6234	-0.13	0.4688
HDL3-apoA-II	0.16	0.3833	-0.13	0.4662	-0.20	0.2703	-0.12	0.4985
HDL4-apoA-II	0.25	0.1834	-0.17	0.3504	-0.23	0.2209	-0.10	0.5667

Spearman correlation analyses were used to evaluate associations of IL-6 with the serum levels of VLDL, IDL, LDL, and HDL. Spearman correlation coefficients with|r|≥0.5 are depicted in bold. ApoA-I, apolipoprotein A-I, apoA-II, apolipoprotein A-II; apoB, apolipoprotein B; C, cholesterol; FC, free cholesterol; HDL, high-density lipoprotein; IDL, intermediate-density lipoprotein; IL-6, interleukin-6; LDL, low-density lipoprotein; MS; metabolic syndrome patient; VLDL, very low-density lipoprotein; PL, phospholipid; TG, triglyceride.
